# PTEN decreases NR2F1 expression to inhibit ciliogenesis during EGFR^L858R^-induced lung cancer progression

**DOI:** 10.1038/s41419-024-06610-z

**Published:** 2024-03-18

**Authors:** Thi Thanh Truc Tran, Jan-Jong Hung

**Affiliations:** Department of Biotechnology and Bioindustry Sciences, National Cheng Kung University, Tainan, Taiwan

**Keywords:** Cancer microenvironment, Cell invasion

## Abstract

Lung cancer is the major cause of death worldwide. Activation of oncogenes or inhibition of tumor suppressors causes cancer formation. Previous studies have indicated that PTEN, as a tumor suppressor, inhibits cancer formation. In this study, we studied the role of PTEN in EGFR^L858R^-induced lung cancer in vivo. Interestingly, loss of PTEN increased bronchial cell hyperplasia but decreased alveolar cell hyperplasia in EGFR^L858R^*PTEN^-/-^-induced lung cancer. Systematic analysis of gene expression by RNA-seq showed that several genes related to ciliogenesis were upregulated in EGFR^L858R^*PTEN^-/-^-induced lung cancer and subsequently showed that bronchial ciliated cells were hyperplastic. Several critical ciliogenesis-related genes, such as *Mucin5A*, *DNAI2*, and *DNAI3*, were found to be regulated by NR2F1. Next, NR2F1 was found to be inhibited by overexpression of PTEN, indicating that PTEN negatively regulates NR2F1, thereby inhibiting the expression of ciliogenesis-related genes and leading to the inhibition of bronchial cell hyperplasia during EGFR^L858R^-induced lung cancer progression. In addition, we also found that PTEN decreased AKT phosphorylation in A549, KRAS mutant, and H1299 cells but increased AKT phosphorylation in PC9, EGFR^L858R^, and H1299^L858R^ cells, suggesting that PTEN may function as a tumor suppressor and an oncogene in lung cancers with KRAS mutation and EGFR mutation, respectively. PTEN acts as a double-edged sword that differentially regulates EGFR^L858R^-induced lung cancer progression in different genomic backgrounds. Understanding the PTEN in lung cancer with different genetic backgrounds will be beneficial for therapy in the future.

## Introduction

Lung cancer, which includes non-small cell lung cancer (NSCLC) and small-cell lung cancer (SCLC), is the leading cause of cancer-related death worldwide. Lung cancer is classified into two main subtypes non-small-cell lung cancer (NSCLC) and small cell lung cancer (SCLC), based on their histological appearance and cellular origin. Epidermal Growth Factor Receptor (EGFR) and Kirsten Rat Sarcoma Viral Oncogene Homologue (KRAS) mutations were associated with the development of NSCLC.

*PTEN*, a well-known tumor suppressor gene, has been reported to be involved in inactivation of the PI3K/AKT signaling pathway, leading to the inhibition of cell adhesion, migration, and invasion. It was observed that the absence of PTEN protein expression resulted in increased phosphorylation of AKT and increased PI3K signaling in the breast cancer cell lines MDA-MB-468 and BT-549 [1]. In lung cancer cells, overexpression of PTEN suppressed cell proliferation and induced cell cycle arrest by reducing the SKP2 protein level [2]. Recent studies have indicated that PTEN may be a biomarker for the response to immunotherapy, suggesting that PTEN regulates not only cancer cells but also other cell types in the tumor microenvironment [3]. Understanding the role and mechanisms of PTEN in cancer progression will be beneficial for the success of cancer therapies using precision medicine.

Ciliated airway epithelial cells have an elongated columnar structure and isolated contact with the basement membrane. Generally, cilia consist of a microtubule, a ciliary membrane, a basal body, and an axoneme [4, 5]. In lung organs, ciliated cells are in bronchioles with club-cells, goblet cells, and basal cells. Previous studies have shown that PTEN interacts with Dishevelled (DVL) to inhibit WNT pathway-mediated ciliogenesis [6]. Recently, studies have indicated that ciliogenesis in the mouse trachea and ependyma requires PTEN [7, 8]. In Xenopus embryos with conditional knockout of PTEN driven by the Foxj1 promoter, ciliogenesis was significantly decreased compared with that in control embryos. However, the role of PTEN in ciliogenesis during cancer progression needs to be clarified. In addition, NR2F1 has been demonstrated to promote cancer cell dormancy in several malignant tumors, thereby leading to recurrence and metastasis during cancer progression [9]. Numerous studies have investigated the role of NR2F1-AS1 as a sponge to inhibit the expression of many miRNAs in turn promote the formation of several cancer [10–12]. In this study, we found that *PTEN* can negatively regulate a transcription factor, NR2F1, to decrease the expression of genes related to ciliogenesis, thereby causing bronchial epithelial cell hyperplasia.

## Results

### Knockout of PTEN enhances bronchial cell growth but inhibits alveolar cell growth in EGFR^L858R^-induced lung cancer

**Fig. 1:**
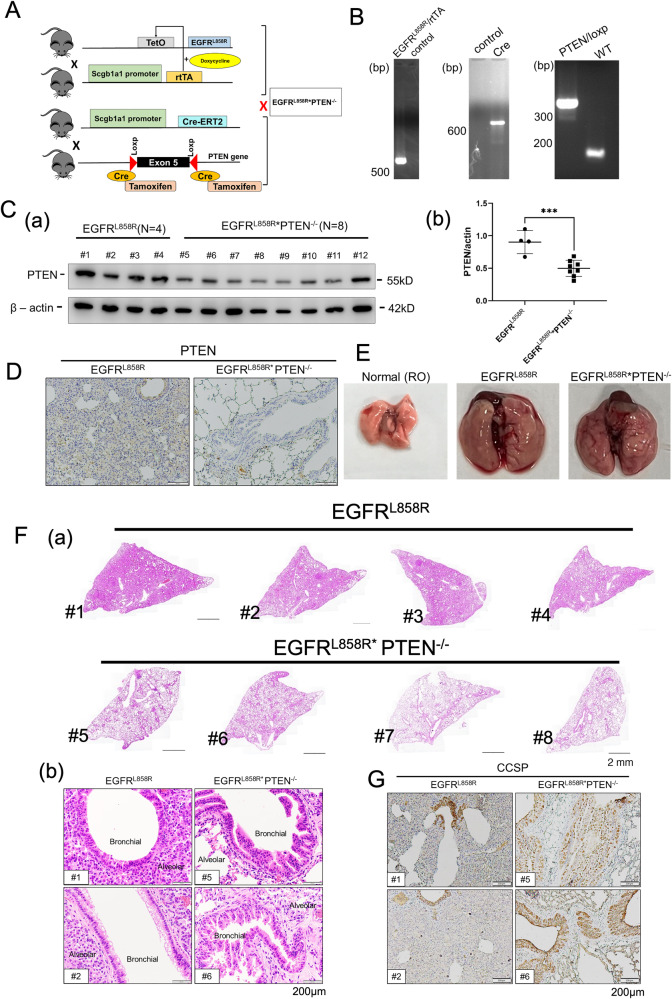
PTEN knockout regulates EGFR^L858R^-induced lung cancer progression. The EGFR^L858R^*PTEN^-/-^ mice were generated by crossing the mice of Tet/EGFR^L858R^/Scgb1a1/rtTA and Scgb1a1/Cre-ERT2/Pten-loxp mice **A**, and the mice were genotyped **B**. The levels of PTEN in the lung tissues of EGFR^L858R^ and EGFR^L858R^*PTEN^-/-^ mice were studied by IB **C** and IHC staining **D** with anti-PTEN antibodies, and then quantitation and statistical analysis were performed; ***p < 0.05. The gross anatomy **E** and H&E staining of EGFR^L858R^ and EGFR^L858R^*PTEN^-/-^ mouse lungs **F** were studied. The levels of CCSP in EGFR^L858R^ and EGFR^L858R^*PTEN^-/-^ mice was evaluated by IHC staining with anti-CCSP antibodies **G**.

PTEN function as a tumor suppressor by inhibiting the PI3K pathway [13]. What is the role of PTEN in bronchial and alveolar cells in EGFR^L858R^-induced lung cancer? In this study, we established an animal model of induced lung cancer, the EGFR^L858R^*PTEN^-/-^ model, in which doxycycline treatment is used to induce the formation of a complex between TetO and scgb1a1-driven rtTA to express EGFR^L858R^ and tamoxifen treatment is used to activate scgb1a1-driven Cre to cut the Lox P sites in the 5^th^ exon of the PTEN gene, namely, its DNA binding motif (Fig. 1A). Genotyping was used to confirm the presence of EGFR^L858R^/rtTA, Cre and Lox P (Fig. 1B). After 2 months of induction by doxycycline treatment, the levels of PTEN in the lungs of EGFR^L858R^*PTEN^-/-^ mice (*n* = 8) were decreased compared to those in the lungs of EGFR^L858R^ mice (*n* = 4) (Fig. 1C, D). The lungs of EGFR^L858R^*PTEN^-/-^ and EGFR^L858R^ mice were larger in size than those of normal (RO) mice (Fig. 1E). In studying lung tissue pathology in EGFR^L858R^*PTEN^-/-^ and EGFR^L858R^ mice, we found that a larger lung area was occupied by cancer cells in EGFR^L858R^ mice than in EGFR^L858R^*PTEN^-/-^ mice (Fig. 1F, a). However, when we evaluated the lung tissues in detail (Fig. 1F, b), we found that knockout of PTEN increased the hyperplasia of bronchial cells but inhibited the growth of alveolar cells in mice with EGFR^L858R^-induced lung cancer, implying a differential effect of PTEN on the tumor burden in bronchial and alveolar cells in mice with EGFR^L858R^-induced lung cancer. Finally, the level of the hyperplasia marker CCSP was measured in EGFR^L858R^*PTEN^-/-^ and EGFR^L858R^ mice (Fig. 1G). The data indicated that the CCSP level was increased, primarily in bronchial cells, in EGFR^L858R^*PTEN^-/-^ mice compared to EGFR^L858R^ mice but was decreased in the alveolar region in the lungs. In summary, PTEN might play different roles in bronchial and alveolar cells in EGFR^L858R^-induced lung cancer.

### Functional knockout of PTEN promotes ciliogenesis in EGFR^L858R^*PTEN^-/-^-induced lung cancer

**Fig. 2:**
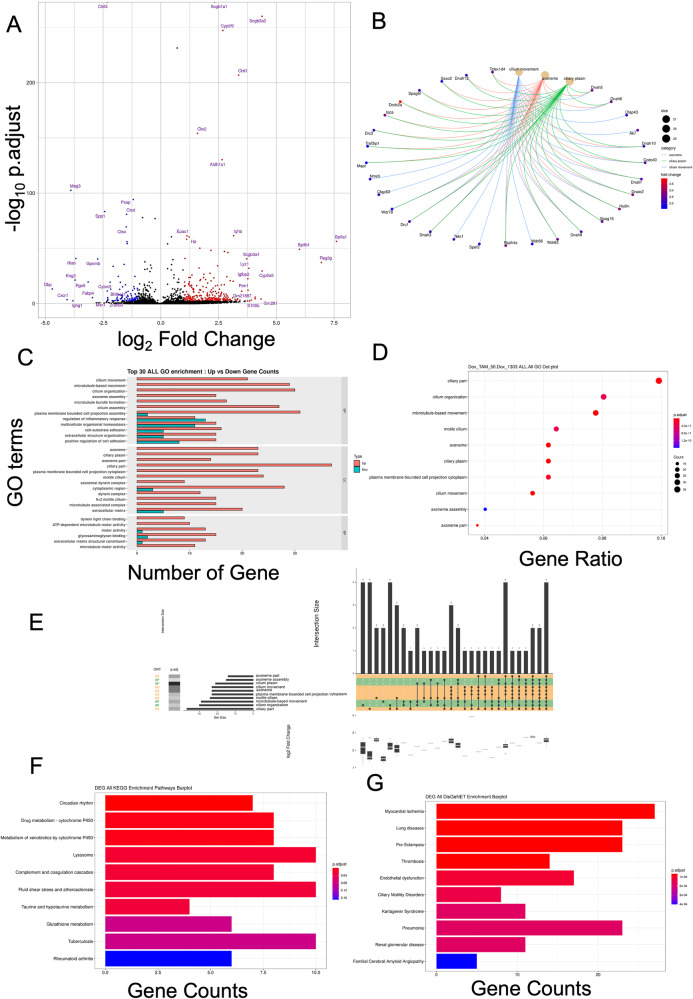
Gene expression profiles in EGFR^L858R^ and EGFR^L858R^*PTEN^-/-^ mice. mRNA was isolated from the lung tissues of EGFR^L858R^ and EGFR^L858R^*PTEN^-/-^ mice for RNA-seq followed by bioinformatics analysis (EGFR^L858R^*PTEN^-/-^/ EGFR^L858R^). **A** Volcano plot; **B** Circus plot; **C** Top 30 terms identified by ALL GO enrichment analysis; **D** ALL GO analysis dot plot; **E** GO analysis Upset plot; **F** Bar plot of DEG All KEGG Enrichment Pathway analysis results; **G** Bar plot of DEG All DisGeNET Enrichment analysis results.

The molecular mechanism by which PTEN regulates bronchial and alveolar cells in EGFR^L858R^-induced lung cancer was next studied (Fig. 2). First, total RNA was isolated from lung tissues of EGFR^L858R^*PTEN^-/-^ and EGFR^L858R^*PTEN^+/+^ mice to systematically study the gene expression, and bioinformatics analysis was then performed (Fig. 2). Quality control indicated that the distribution of gene expression was similar between EGFR^L858R^*PTEN^-/-^ and EGFR^L858R^*PTEN^+/+^ mice, suggesting that the RNA-Seq data were of good quality (Suppl. Fig. 1). In PTEN knockout mice, 288 genes were upregulated, and 97 genes were downregulated in EGFR^L858R^-induced lung cancer tissues (Fig. 2A). In the Circus plot, most of the PTEN-regulated genes can be seen to be involved in three pathways, namely, ciliary plasm, axoneme and cilium movement, implying that PTEN might regulate ciliated bronchial cells in EGFR^L858R^-induced lung cancer (Fig. 2B and Supplementary Table 1). The list of the top 30 terms identified ALL GO enrichment analysis shows that PTEN was involved in most of the pathways, such as cilium movement, microtubule-based movement, and cilium organization, indicating that PTEN inhibits hyperplasia of ciliated cells in EGFR^L858R^-induced lung cancer (Fig. 2C). In addition, based on statistical significance, the dot plot of the ALL_GO analysis results revealed that knockout of PTEN resulted in enrichment of pathways related to ciliary organization and movement (Fig. 2D). Next, we also used Upset analysis to study the related genes in the different pathways (Fig. 2E). The data indicated that several genes were involved in most of the pathways related to cilium organization and movement (Fig. 2E). Next, the bar plot of the DEG All KEGG Enrichment Pathways analysis results suggested that loss of PTEN activated several pathways involved in circadian rhythms, drug metabolism, metabolism of xenobiotics, lysosomes and so on (Fig. 2F). Finally, the bar plot of the DEG All DisGeNET Enrichment analysis results indicated that loss of PTEN positively regulated myocardial ischaemia, lung diseases, preeclampsia, thrombosis, endothelial dysfunction and so on (Fig. 2G). In summary, loss of PTEN positively regulates several genes related to ciliogenesis, which may increase the hyperplasia of bronchial ciliated cells in EGFR^L858R^-induced lung cancer.

**Fig. 3:**
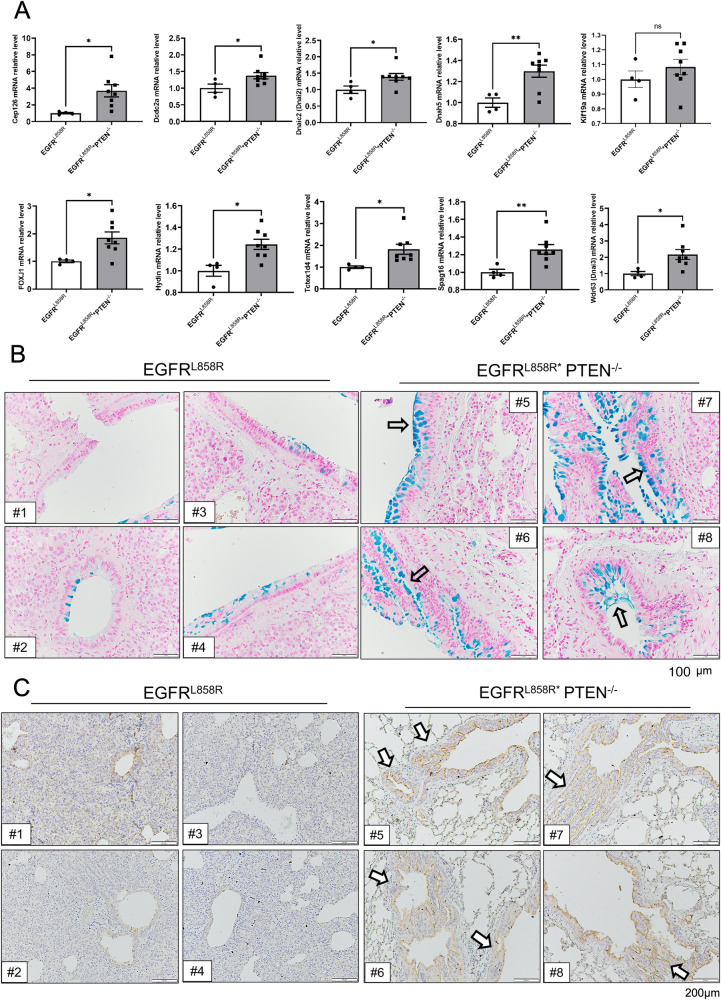
PTEN knockout induces hyperplasia of bronchial epithelial cells. mRNA isolated from the lungs of EGFR^L858R^ and EGFR^L858R^*PTEN^-/-^ mice was used to measure the levels of cilia-related genes by qPCR, and then statistical analysis was performed by a t test; *p < 0.05, **p < 0.01, ***p < 0.001 **A**. The ciliated cells on the surface of bronchial cells were studied by Alcian blue staining **B** and IHC staining with anti-mucin antibodies **C**.

Next, we prepared more mice to study the effect of PTEN on these cilia-related genes (Fig. 3). Indeed, the results indicated that PTEN knockout significantly increased the mRNA levels of most of the ciliated genes, namely, *Cep126*, *Dcdc2a*, *Dnaic2*, *Dnah5*, *Foxj1*, *Hydin*, *Tctex1d4*, *Spag16*, and *Wdr63*, implying that loss of PTEN may be involved in ciliogenesis during EGFR^L858R^-induced lung cancer progression (Fig. 3A). Next, we used Alcian blue staining to study the ciliated cells among bronchial epithelial cells from mice with EGFR^L858R^*PTEN^+/+^- and EGFR^L858R^*PTEN^-/-^-induced lung cancer (Fig. 3B). The data indicated that PTEN knockout strongly increased the Alcian blue signal in bronchial epithelial cells, suggesting that loss of PTEN stimulates hyperplasia of bronchial epithelial cells in the EGFR^L858R^*PTEN^-/-^ mouse model of lung cancer (Fig. 3B). Finally, we also used a marker of cilia, acetylated tubulin, to study the effect of PTEN on ciliogenesis in EGFR^L858R^-induced lung cancer (Fig. 3C). The data indicated that acetylated tubulin was more abundant in the bronchial of EGFR^L858R^*PTEN^-/-^ mice compared to EGFR^L858R^ mice (Fig. 3C). In summary, PTEN inhibits most of cilia-related genes to regulate ciliogenesis, thereby suppressing bronchial epithelial cell hyperplasia in EGFR^L858R^-induced cancer.

### PTEN inhibits NR2F1 expression to decrease ciliogenesis-related gene expression in EGFR^L858R^-induced lung cancer

**Fig. 4:**
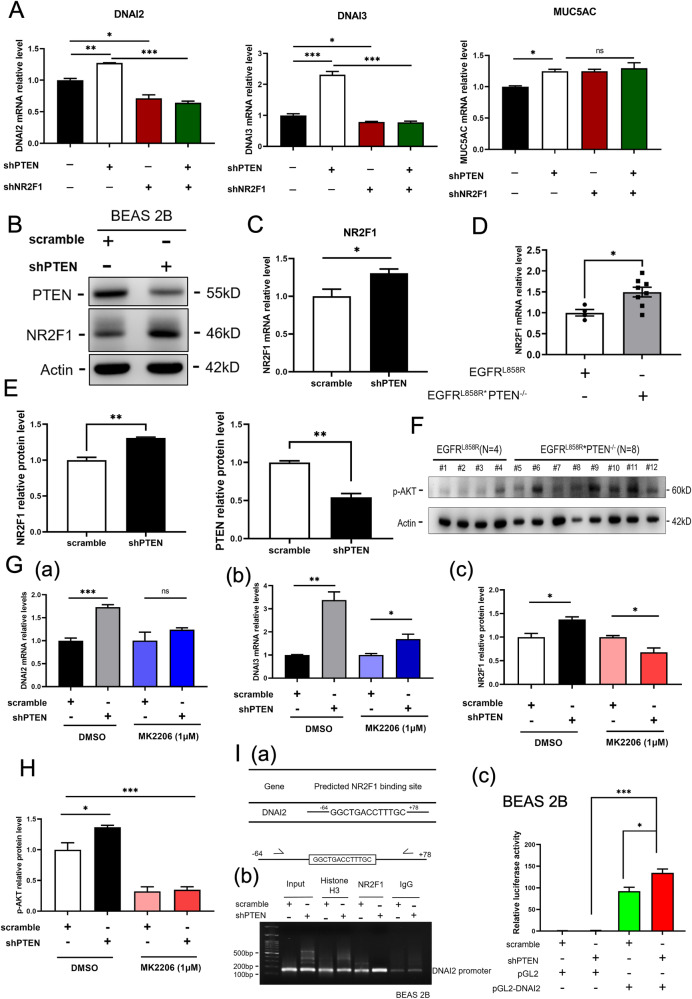
PTEN decreases NR2F1 expression to inhibit DNAI2 expression in lung cancer. mRNA levels of DNAI2, DNAI3 and MUC5AC in BEAS 2B cells with or without knockdown of PTEN and NR2F1 were measured by qPCR **A**. The levels of proteins **B** and mRNAs **C** in BEAS 2B cells with or without knockdown of PTEN and NR2F1 were measured by IB and qPCR. The mRNA levels of NR2F1 in the lungs of EGFR^L858R^ and EGFR^L858R^*PTEN^-/-^ mice and in PTEN knockdown BEAS 2B cells were measured by qPCR **D**, **E**. The p-AKT levels in the lungs of EGFR^L858R^ and EGFR^L858R^*PTEN^-/-^ mice were evaluated by IB **F**. The mRNA levels of DNAI2 **G**, a and DNAI3 **G**, b in BEAS 2B cells with or without PTEN knockdown and MK2206 treatment were measured by qPCR. The protein levels of NR2F1 **G**, c and p-AKT **H** in BEAS 2B cells with or without PTEN knockdown and MK2206 treatment were measured by IB. The recruitment of NR2F1 to the promoter of DNAI2 in BEAS 2B cells was evaluated by a chromatin immunoprecipitation (ChIP) assay **H** and (I, a, b). The transcriptional activity driven by the promoter of NR2F1 (2 kb) with or without PTEN knockdown was measured by luciferase activity assay I, c. The statistical analysis was performed by a t test; *p < 0.05, **p < 0.01, ***p < 0.001, ns: nonsignificant.

Next, we studied how PTEN regulates the expression of cilia-related genes (Fig. 4). We investigated the transcription factors possibly recruited to the promoters of all the PTEN-regulated cilia-related genes and then checked the read numbers of these transcription factors in the RNA-seq data. The results showed that NR2F1 can be recruited to the promoters of most cilia-related genes and increased their read numbers in mice with EGFR^L858R^*PTEN^-/-^-induced lung cancer, implying that PTEN may inhibit NR2F1 to in turn inhibit the expression of cilia-related genes. First, we examined the effect of PTEN on the expression of cilia-related genes in BEAS 2B (Fig. 4A). The data indicated that PTEN knockdown increased the mRNA levels of DNAI2, DNAI3, and MUC5AC in BEAS 2B cells, a bronchial cell line. Knockdown of NR2F1 abolished the effect of PTEN on DNAI2 and DNAI3 expression but not MUC5AC expression (Fig. 4A). Next, the protein and mRNA levels of NR2F1 were found to be increased in PTEN knockdown BEAS 2B cells (Fig. 4B, C). The NR2F1 level was also increased in EGFR^L858R^*PTEN^-/-^ mice compared to that of EGFR^L858R^-mice (Fig. 4D) and in PTEN-silenced BEAS 2B cells (Fig. 4E), indicating that PTEN negatively regulates NR2F1 in vitro and in vivo. PTEN negatively regulates the AKT signaling pathway, which is involved in cancer progression [14]. Thus, knockout of PTEN increased the levels of phosphor-AKT in EGFR^L858R^*PTEN^-/-^ mice compared to EGFR^L858R^ mice (Fig. 4F). Additionally, inhibition of AKT phosphorylation by MK2206 treatment abolished the effect of PTEN on the expression of DNAI2, DNAI3 and NR2F1 (Fig. 4G, H). Finally, loss of PTEN increased the recruitment of NR2F1 to the promoter of DNAI2 (Fig. 4I, a and b) and increased the luciferase activity driven by the promoter of NR2F1 (2 K bp) (Fig. 4I, c), indicating that NR2F1 can regulate DNAI2 directly. In summary, PTEN suppresses AKT activation, thereby inhibiting NR2F1 expression and subsequently decreasing cilia-related gene expression, leading to inhibition of bronchial epithelial hyperplasia in EGFR^L858R^-induced lung cancer.

### PTEN promotes alveolar cell growth in EGFR^L858R^-induced lung cancer

**Fig. 5:**
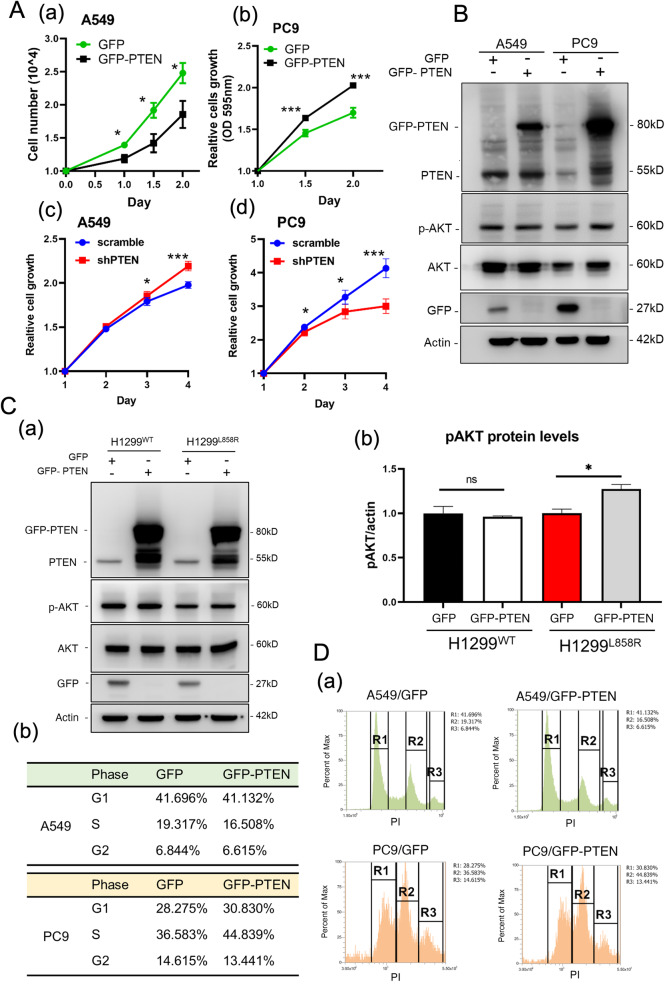
PTEN promotes the growth of alveolar cells with EGFR mutation. The role of PTEN in the cell growth of A549 and PC9 cells with or without GFP-PTEN overexpression or PTEN knockdown was evaluated by cell counting **A**. The levels of phosphor-AKT (p-Akt) in A549, PC9 **B**, H1299^WT^ and H1299^L858R^ **C** cells with or without GFP-PTEN overexpression were measured by IB with antibodies against the indicated proteins. The effect of PTEN on the cell cycles in A549 and PC9 cells with or without GFP-PTEN overexpression was evaluated by flow cytometry **D**. The statistical analysis was performed by a t-test; *p < 0.05, **p < 0.01, ***p < 0.001, ns: nonsignificant.

Thus far, we have elucidated the mechanism by which PTEN negatively regulates hyperplasia of bronchial cells in EGFR^L858R^-induced lung cancer. However, what is the molecular mechanism by which PTEN positively regulates alveolar cellular growth in EGFR^L858R^-induced lung cancer (Fig. 5)? In addition, is the positive effect of PTEN in EGFR^L858R^ mice dependent? We used two alveolar lung cancer cell lines, A549 and PC9, which have KRAS and EGFR mutations, respectively, to study the role of PTEN in cell proliferation (Fig. 5A). Overexpression of GFP-PTEN inhibited A549 cell growth but promoted PC9 cell growth (Fig. 5A, a and b). Knockdown of PTEN increased A549 cell growth and decreased PC9 cell growth (Fig. 5A, c and d), indicating that PTEN negatively regulates KRAS-mutant cell growth but positively regulates EGFR^L858R^-mutant cell growth. In addition, overexpression of GFP-PTEN increased the level of phosphorylated AKT (p-AKT) in PC9 and H1299^L858R^ cells but not in A549 and H1299 cells, suggesting that the EGFR^L858R^ mutation is critical for the effect of PTEN on alveolar cell growth (Fig. 5B, C). Finally, the cell cycles in PC9 and A549 cells with or without GFP-PTEN overexpression was studied by flow cytometry (Fig. 5D). The data indicated that overexpression of GFP-PTEN increased the percentage of G1/S-phase PC9 cells (64.9%/75.7%) but decreased the percentage of G1/S-phase A549 cells (61.0%/57.6%), indicating that PTEN may have differential effects on alveolar cells with different mutation backgrounds, a finding that is expected to be beneficial for precision therapy in the future (Fig. 5D). In summary, differential effects of PTEN were found on EGFR-mut and KRAS-mut lung cancer cell lines, implying that the PTEN status might be important for clinical lung cancer patients with EGFR mutations or KRAS mutations.

### The clinical relationships between the survival rate and the expression of PTEN and ciliogenesis-related proteins

**Fig. 6:**
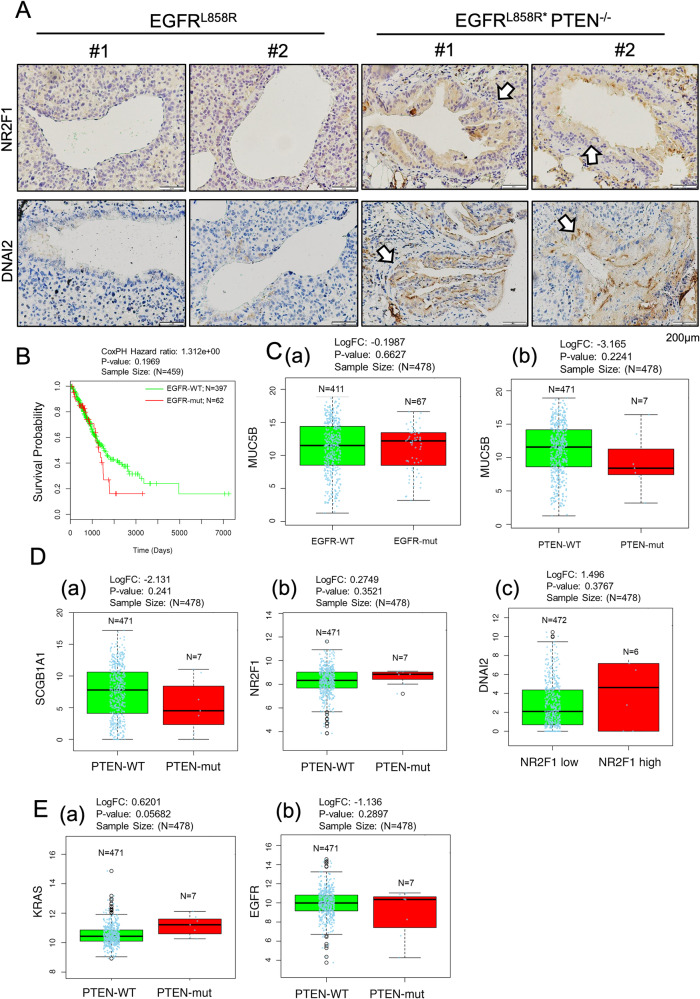
The relationships between the survival rate and the expression of cilia-related proteins in lung cancer. The levels of NR2F1 and DNAI2 in EGFR^L858R^*PTEN^+/+^ and EGFR^L858R^*PTEN^-/-^ mice were evaluated by IHC staining **A**. The relationship between the survival probability and EGFR-WT/EGFR-mut status in the lung cancer cohorts from TCGA was studied **B**. The correlations between MUC5B and EGFR-WT/EGFR-mut **C**, a, MUC5B and PTEN-WT/PTEN-mut **C**, b, SCGB1A1 and PTEN-WT/PTEN-mut **D**, a, NR2F1 and PTEN-WT/PTEN-mut **D**, b, DNAI2 and negative NR2F1/positive NR2F1 **D**, c, KRAS and PTEN-WT/PTEN-mut **E**, a, and EGFR and PTEN-WT/PTEN-mut **E**, b in the lung cancer cohorts from TCGA were studied.

**Fig. 7:**
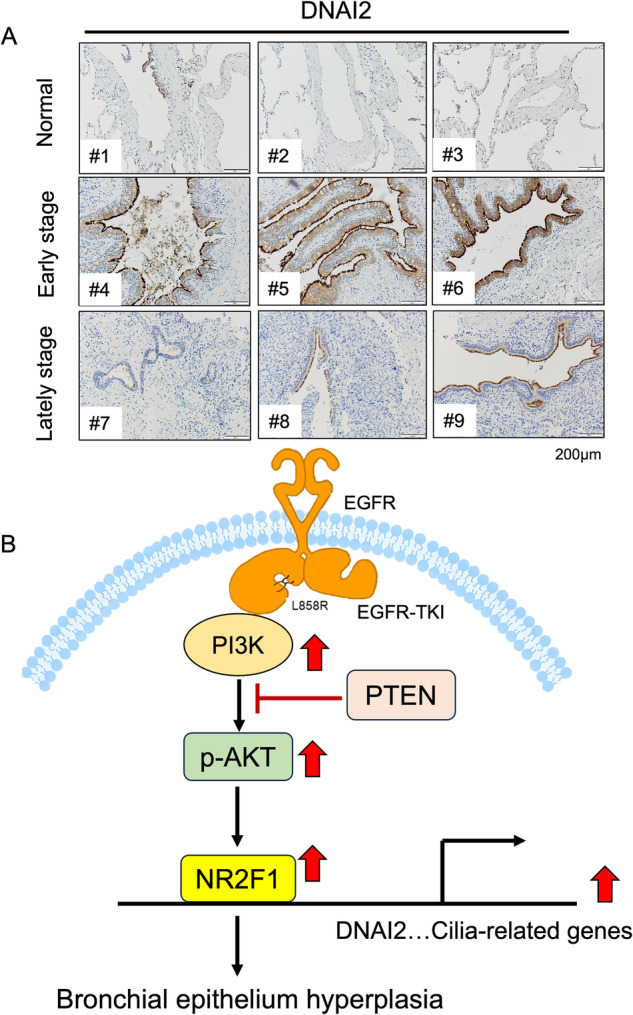
The level of DNAI2 in lung cancer patients. The level of DNAI2 in the early and late stages of lung cancer in the indicated cohorts was evaluated by IHC staining with an anti-DNAI2 antibodies **A**. Working model: PTEN inhibits NR2F1 expression to decrease bronchial cell hyperplasia during EGFR^L858R^-induced lung cancer progression but does not have this effect on alveolar cells **B**.

Next, we used online data, animal samples and clinical specimens to study the relationship among survival, PTEN, NR2F1 and cilia-related proteins (Figs. 6 and 7). First, the levels of NR2F1 and DNAI2 in EGFR^L858R^*PTEN^-/-^ and EGFR^L858R^*PTEN^+/+^ mice were measured (Fig. 6A). The data indicated that the levels of both NR2F1 and DNAI2 were dramatically higher in EGFR^L858R^*PTEN^-/-^ mice, implying that loss of PTEN increases NR2F1 expression, thereby promoting DNAI2 expression and leading to cilium formation (Fig. 6A). Next, the related information of non-small cell lung cancer (NSCLC) cohorts from TCGA was used to study the relationships between the survival rate and the expression of cilium-related genes (Fig. 6B–E). First, the survival rate in the lung cancer cohorts with EGFR mutations was found to be lower than that in the cohort with wild-type EGFR (Fig. 6B). MUC5B expression was decreased in the EGFR-mut and PTEN-mut cohorts (Fig. 6C). In addition, SCGB1A1 expression was also inhibited in the PTEN-mut cohort (Fig. 6D, a). NR2F1 was highly expressed in the PTEN-mut cohorts (Fig. 6D, b). There was a positive correlation between NR2F1 and DNAI2 expression in all the NSCLC patients (Fig. 6D, c). Finally, the KRAS level was different in the PTEN-WT and PTEN-mut cohorts, but no difference was found in the EGFR level, implying that the effect of PTEN on KRAS-mutant and EGFR-mutant cancers is different (Fig. 6E).

Finally, we collected lung cancer clinical specimens to compare the level of DNAI2 in tissues of different stages (Fig. 7A). The results indicated that DNAI2 was increased in the early stage of NSCLC but was decreased in the late stage, indicating that DNAI2 might be involved in lung tumorigenesis (Fig. 7A).

## Discussion

In this study, we found that loss of PTEN increases NR2F1 expression and subsequently increases DNAI2 expression, leading to bronchial cell hyperplasia but inhibiting alveolar cell hyperplasia during EGFR^L858R^-induced lung cancer progression, a finding expected to be beneficial for the precision medicine in the treatment of lung cancer in the future (Fig. 7B).

Interestingly, PTEN is well known as a tumor suppressor in the various cancer types. However, we found that PTEN may act as a double-edged sword in lung cancer progression. In this study, we found that PTEN knockout induced the hyperplasia of bronchial epithelial cells but inhibited the growth of alveolar cells in EGFR^L858R^-induced lung cancer. Previous studies revealed that PTEN knockout also induced the hyperplasia of bronchial epithelial cells but did not change the growth of alveolar epithelial cells in KRAS^G12D^-induced lung cancer, in which PTEN inactivation cooperated with oncogenic KRAS^G12D^ to promote lung cancer progression [15]. First, loss of PTEN was found to increase the hyperplasia of bronchial epithelial cells in EGFR^L858R^- and KRAS^G12D^-induced lung cancer. In this study, we clarified that PTEN negatively regulates NR2F1 to inhibit cilia-related gene expression in EGFR^L858R^-induced lung cancer, a mechanism that is involved in bronchial epithelial cell hyperplasia. In addition, numerous studies have indicated that NR2F1-AS1 sponges many miRNAs, subsequently inducing the expression of several oncogenes [10–12]. Therefore, PTEN may also inhibit NR2F1-AS1 to suppress lung cancer progression. In addition, previous studies have indicated that NR2F1 may regulate dormancy and metastasis in the late stage of lung cancer [16, 17]. In this study, we used EGFR^L858R^ mice to study the initiation of lung cancer, not the late stage of lung cancer. Based on previous studies and this study, NR2F1 may have differential effects on cancer in the early and late stages. In addition, although PTEN also induces hyperplasia in bronchial epithelial cells of KRAS^G12D^-induced lung cancer mice [15], the molecular mechanism is not yet clarified. Second, PTEN plays different roles in bronchial and alveolar epithelial cells of mice with EGFR^L858R^-induced lung cancer but not in mice with KRAS^G12D^-induced lung cancer. This is the first study to identify the dual roles of PTEN as a tumor suppressor and an oncogene in bronchial and alveolar epithelial cells, respectively, in EGFR^L858R^-induced lung cancer. Overexpression of PTEN increased cell growth in the EGFR-mutant cell lines PC9 and H1299^L858R^ but not in the KRAS-mutant cell line A549, indicating that EGFR mutation is critical for PTEN-mediated cell growth. In analyzing the RNA-seq data, we found that PTEN knockout decreased Akt1 and Akt2 expression but increased Akt3 expression (data not shown). Previous studies also indicated that, unlike Akt1 and Akt2 phosphorylation, Akt3 phosphorylation inhibits cancer cell proliferation [18, 19]. However, why the EGFR mutation is required for the enhancement of cell proliferation by PTEN in alveolar cells needs to be addressed in the future. Finally, EGFR is one of the most well-known tyrosine kinase receptor domain-containing proteins and is commonly mutated in tumor, with uncontrolled cell growth, proliferation, and migration documented in approximately 33% of NSCLCs [20, 21]. The five-residue deletion (746ELREA750) in exon 19 accounts for 47% of EGFR mutations, and the exon 21 (L858R) substitution accounts for 41% [22]. Herein, we used mice with EGFR^L858R^-induced lung cancer and cancer cells to study the role of PTEN in bronchial and alveolar cells. Whether other EGFR mutation types also have the same effects needs to be clarified in the future.

In this study, we found that loss of PTEN significantly induced the proliferation of ciliated cells among bronchial epithelial cells of mice with EGFR^L858R^-induced lung cancer. Furthermore, we clearly clarified the molecular mechanism by which PTEN regulates ciliogenesis in bronchial epithelial cells during lung cancer progression. During lung morphogenesis, the ciliated cell differentiation pathway is activated [23, 24]. Forkhead box protein J1 (Foxj1), a transcription factor required for cilia formation and motility, is also expressed [25, 26]. Patients with Chronic obstructive pulmonary disease (COPD) has a considerable reduction in the number of ciliated cells [27, 28]. Ciliated cell dysfunction not only causes this disorder but is also found in various cancers. A primary cilium is frequently present in cells of human differentiated thyroid tumors [29, 30]. The results were indicated that the lack of primary cilia resulted in increased apoptosis in thyroid cancer cells, possibly reveling a new therapeutic target for thyroid cancers [30]. In addition, primary cilia can be formed on pancreatic cancer cells and that their presence is strongly correlated with the prognosis of pancreatic ductal adenocarcinoma [31, 32]. In contrast, in oral squamous cell carcinoma (OSCC), a significant reduction in the percentage of ciliated cells was found in oral leukoplakia (OLK), especially in patients with OSCC, and EGFR was a target, suggesting that loss of cilia induced oral tumor growth [33]. Because PTEN is a famous tumor suppressor that regulates the expression of numerous genes involved in cell biogenesis, the link between PTEN and cilia needs to be discussed. However, another study showed the conflicting result that PTEN negatively regulates dendritogenesis. Loss of PTEN is related to autism spectrum disease (ASD) and causes excessive neuronal development, including the formation of lengthened and branched total dendritic spines [34]. Ciliated cells are progeny of club cells, such as goblet cells, and a complicated transcriptional network including Notch signaling controls how these cells differentiate [35, 36]. Mucins are glycoproteins that are secreted by gel-forming mucin-producing goblet cells.

In this study, we found that PTEN knockout induced mucin-related genes. Two high-molecular-weight secreted mucins that are expressed mainly in the mucus layer, which contains electrolytes, metabolites, fluids, and antimicrobial substances, are MUC5AC and MUC5B [25, 37]. The mucus layer serves as the first line of innate protection in the respiratory tract against inhaled pathogens and particles [38–40]. Ciliated cells facilitate mucin trafficking to trap particles and as components of the ciliary escalator that drives mucus into the oropharynx for eventual removal by expectoration or swallowing. PTEN regulates mucin-related genes but not through NR2F1. The detailed mechanism by which PTEN regulates mucin gene expression will be addressed in the future.

Dynein Axonemal Intermediate Chain 2 (DNAI2) is part of the dynein complex of respiratory cilia and sperm flagella [41, 42]. Mutations in DNAI2 are involved in the development of primary ciliary dyskinesia type 9. Recent studies have also shown that various isoforms encoded by alternatively spliced transcript variants are involved in primary ciliary dyskinesia (PCD), which may be as a new genetic risk factor for PCD [43, 44]. There is no reported study about DNAI2 in cancer. This is the first study to determine the role of PTEN-mediated DNAI2 expression in the hyperplasia of bronchial epithelial cells in EGFR^L858R^-induced lung cancer. Recent studies have also revealed that PTEN mutations might be involved in mediating drug resistance and immunotherapy efficacy during cancer therapy [45–47]. PTEN mutation-induced ciliogenesis in bronchial epithelial cells might be a critical mechanism. In addition to cilia-related genes, other PTEN-regulated genes were also found in our RNA-seq analysis. For example, ACE2, which has been reported to be overexpressed in different cell subsets of NSCLC, was also upregulated in EGFR^L858R^*PTEN^-/-^ mice [48, 49]. In addition, previous studies indicated that PTEN inactivation in mice with Kras^G12D^-induced lung cancer increased the immune response [15, 50, 51]. In this study, we also found that several genes related to the inflammatory response, lymphocyte activation and innate immune response in the mucosa (*Hp/Reg3g/Nupr1/Adam8/Cd55/Cfh/Ednrb*) were upregulated in mice with EGFR^L858R^*PTEN^-/-^ induced lung cancer. Finally, many genes related to epithelial cell migration, extracellular matrix assembly and cellular extravasation were upregulated by PTEN knockout, implying that PTEN may significantly promote cancer metastasis. The effect of the PTEN-regulated tumor microenvironment on cancer metastasis in vivo might be evaluated in vivo by using models of EGFR^L858R^- or KRAS^G12D^-induced lung cancer in the future.

## Materials and methods

### Cell culture and reagents

Human lung bronchial epithelial cell lines BEAS-2B and adenocarcinoma human alveolar basal epithelial cells A549, PC9 were cultured with RPMI 1640 medium (Invitrogen, Carlsbad, CA, USA) containing 10% fetal bovine serum (FBS) (Gibco™, Waltham, MA, USA), 100 µg/ml streptomycin and 100 U/ml penicillin G (Gibco). All cells were maintained in a humidified incubator under 5% CO2 at 37 ^o^C. For AKT inhibitor treatment, cells were seeded until reached confluence and treated with 1 µM MK-2206 (Merk Millipore Corp, Billerica, MA, USA) for 24 h. For transfecting plasmid, PolyJet (SignaGen Laboratories, Frederick, MD, USA) was used according to the manufacturer’s instructions.

### Alcian blue staining

The paraffin embedded sections were incubated in xylene for dewaxing and a graded series of ethanol for hydration. Next, sections were incubated in 3% acetic acid for 5 min and stained by Alcian blue (pH = 2.5) (Merk Millipore Corp, Billerica, MA, USA) solution for 30 min at room temperature. To remove exceed Alcian Blue and prevent non-specific staining, slides were rinsed briefly by 3% acetic acid for 10 seconds. The tissue samples were rinsed two times with distilled water and stained with 0.1% Nuclear Fast Red solution (Merk Millipore Corp, Billerica, MA, USA) for 5 min to visualize histologic changes. All slices were dehydrated through graded alcohols before mounting.

### Animal study

Transgenic mice (B6 strain) were acquired from Jackson Lab and maintained at the National Laboratory Animal Center in Taiwan. Reverse tetracycline trans-activator (rtTA) protein was expressed under control of Scgb1a1 (secretoglobin, family 1 A, member 1) promoter in Scgb1a1-rtTA transgenic mice. To generate the EGFR mutation, the TetO-EGFR^L858R^ transgenic mice, tetracycline- responsive promoter element (TRE; tetO) was utilized. TetO-EGFR^L858R^ mice were crossed with Scgb1a1-rtTA transgenic mice to generate bi-transgenic mice. After breeding Scgb1a1-rtTA/TetO-EGFR^L858R^ mice were used to study lung cancer. In order to induce lung cancer progression, doxycycline (0.5 g/l) was dissolved in drinking water for two-month-old transgenic mice orally administration. To engineer PTEN conditionally knockout mice, two-month-old transgenic mice were administrated with intraperitoneal tamoxifen injection (1 mg/kg) and combined with tamoxifen in food (400 mg/kg) for one month. All mice were continuously maintained for two months and were sacrificed. Lung tissues were analyzed by immunohistochemistry staining. All treatments involving animals were conducted in accordance with the rules and regulations of Institutional Animal Care and Use Committee at National Cheng Kung University.

### RT-PCR and q-PCR

Total RNA of cells was isolated with a TRizol RNA extraction kit (Invitrogen, Waltham, MA, USA) and 2 µg of RNA was subjected to RT-PCR with SuperScript II enzyme (Invitrogen, Waltham, MA, USA). cDNA was synthesized using RT-PCR program. Next, the cDNA products were determined the cycle-threshold (Ct) fluorescence value by using SYBR Premix Ex Tag in CFX96TM Real-Time System and C1000 TM Thermal Cycler. The primers for quantitative PCR were shown in Supplementary Table 2. The mRNA level of each gene was presented using 2-ΔCT, where ΔCT = (Ct Gene - Ct GAPDH). GAPDH as internal control.

### Statistical analysis

All samples were used for statistical analysis. Data was analyzed using the GraphPad Prism software and western blot images were calculated by ImageJ software. The difference between two groups was analyzed by Student’s t test: *p < 0.05, **p < 0.01, ***p < 0.001. The data analysis would be considered statistically significant if p value is lower than 0.05.

### Supplementary information

Supplemental Information (merge version)

Original Data File

Reproducibility checklist

## Data Availability

All data generated or analyzed during this study are included in this published article and its supplementary information files. The datasets generated and/ or analyzed during the current study are available from the corresponding author upon reasonable request.
