# Supplementary material for: PTEN decreases NR2F1 expression to inhibit ciliogenesis during EGFRL858R-induced lung cancer progression
Source: Cell Death Dis. 2024 Mar 18;15(3):225. doi: 10.1038/s41419-024-06610-z (PMC10948910; doi:10.1038/s41419-024-06610-z)
Supplement: Supplementary file 1 — Supplemental Information (merge version) [file 41419_2024_6610_MOESM1_ESM.docx]

**Supplementary information**

**Supplementary Materials and Methods**

**Lentivirus knockdown system** - The lentivirus-scramble, lentivirus-PTEN-shRNA and lenti-NR2F1-shRNA viruses were generated from the National RNAi core Facility (Academia Sinica, Taipei, Taiwan). Cells were seeded in 6-well-plates and incubated for 16 hours, and then treated with 1ml RPMI medium (Invitrogen, Carlsbad, CA, USA) containing 10 µg Polybrene (Merk Millipore, Darmstadt, Germany) and lentivirus with 10 multiplicity of infection (MOI). After 24 hours of infection, medium containing lentivirus was replaced with fresh medium and maintained for another 72 hours.

**Western blotting -** Cell lysates were prepared from the indicated cell lines for SDS-polyacrylamide gel electrophoresis (SDS-PAGE), which was then transferred to a PVDF membrane by using a transfer apparatus according to the manufacturer’s protocols. Membranes were blocked with 5% nonfat milk in TBST buffer (10mM Tris-HCL, pH 8.0, 150mM NaCl and 0.05% Tween 20) for 1h, washed in the same buffer and incubated with antibodies against PTEN (1:1000) (Cell Signaling, Danvers, MA, USA), NR2F1 (1:1000) (Abcam, Cambridge, United Kingdom ), p-AKT (1:1000) (Cell Signaling, Danvers, MA, USA), AKT (1:1000) (Cell Signaling, Danvers, MA, USA), β actin (1:10000) (Cell Signaling, Danvers, MA, USA) at 4^o^C overnight. Membranes were washed three times for 10 min and incubated with the secondary antibody (goat-anti rabbit or anti- mouse immunoglobulin G linked with horse radish peroxidase) for 1h at room temperature. After three more washes, the protein bands were detected with the ECL Western blotting detection System (Merk Millipore Corp, Billerica, MA, USA) and recorded with the FlourChem image analysis system. Band intensities were quantified with ImageJ software.

**Luciferase reporter assay-** 8x10^4^ BEAS-2B cells were seeded in each well of 6-well plates for 16 hours. Reporter plasmids containing DNAI2 promoter region were transfected into BEAS-2B cells. Reporter assays were performed by using Dual-luciferase reporter assay system (Promega, Madison, USA) following manufacturer’s instruction. All the primers used in this study for plasmid construction and RT-PCR were listed in Supplementary Table 2.

**Plasmid construction** - To construct a 2kb promoter of DNAI2, genomic DNA from BEAS-2B was extracted. The DNAI2 promoter (2kb) was generated by PCR using primers: F, 5’-GGT ACC TAA CCC ACT TTC CCA CCC ACA A -3’ and R, 5’- GCT AGC ACG TGA CTC CTT TCC TGC CAC -3’. After amplification and purification, the DNA fragments were ligated to yT&A (Yeastern Biotech Co., Ltd., Taipei, Taiwan). To confirm successful ligation, blue- white screening was performed with IPTG/X-gal selection. The ligation products were transformed into competent cell DH5α (Real Biotech Corporation, Taipei, Taiwan) and incubated at 37^o^C for 16h. The transformed cells with vector containing DNA product will show as white colony. Then, 2kb promoter of DNAI2 was continuously ligated into pGL2 vector using restriction enzyme KpnI and NheI (New England Biolabs, Ipswith, MA, USA). Plate competent cells containing pGL2-DNAI2-2kb in LB medium which were selected by ampicillin at 37^o^C for 16h. The completed plasmids were isolated using a Gene- Spin^TM^ MiniPrep Purification Kit (Protech System, Taipei, Taiwan) and checked sequencing by Genomic®.

**Immunohistochemistry (IHC)** - Paraffin-embedded human and mice lung cancer tissues were obtained from the National Cheng Kung University Hospital Tissue Bank and doxycycline-induced EGFR^L858R^ mice or EGFR^L858R^*PTEN^-/-^-induced lung cancer mice, and the tissues were cut into 5-μm sections. Immunohistochemistry was performed using a Novolink™ Polymer Detection Systems (Leica Biosystems) following the manufacturer’s instructions. Antigen retrieval was performed using citrate buffer (pH 6.0, Scytek). Primary antibodies, anti-PTEN (138G6, Cell Signaling, 1:100), anti-NR2F1 (sc-74560, Santa Cruz Biotechnology, Inc, 1:100), anti-CCSP (A16997, ABclonal, 1:100), anti-MUC5AC (A17325, ABclonal 1:100) and anti-DNAI2 (17533-1-AP, Proteintech, 1:100), were used to incubate with tissue samples for overnight at 4 °C. Sections were photographed by Olympus BX-51 microscope.

**Chromatin immunoprecipitation (ChIP)** - BEAS 2B cells were infected with scramble or shPTEN shRNA expressing lentivirus and added 37% formaldehyde into medium to a final concentration of 1% at room temperature for 10 minutes. Cells were continuously added glycine at final concentration of 125mM to stop cross-linking. All cells were washed with PBS and resuspended with ChIP lysis buffer (pH 8 1M Tris-HCl, 150mM NaCl, 5mM EDTA, 1% Triton X-100, 1% SDS, 1% NaDOC). Samples were sonicated (amplitude 50%, 15 seconds on, 15 seconds off, total 10 minutes) on ice to shear chromatin to an average length between 300 to 5000 bps, and the supernatants were collected by centrifugation with 8000rpm, 10 minutes at 4^o^C. Supernatant were the diluted five-time with ChIP lysis buffer and NR2F1 antibody (1:200), IgG (1:200). Histone H3 (1:200) were added. Samples were incubated at 4^o^C on a rotating device overnight, then 50 µl of Protein G Plus/Protein A-Agarose (Merk Millipore Corp, Billerica, MA, USA) was added and incubated more 6 hours. Agarose beads were collected by centrifuge at 10.000 rpm for 5 minutes and wash with low salt buffer (1% SDS, Triton X-100, 5mM EDTA, pH 8 1M Tris-HCl, 150mM NaCl) for two times, high salt buffer ((1% SDS, Triton X-100, 5mM EDTA, pH 8 1M Tris-HCl, 500mM NaCl) for two times, LiCl buffer (250mM LiCl, 1% NP-40, 1% NaDOC, 5mM EDTA, pH 8 1M Tris-HCl) for two times and TE buffer (5mM EDTA, pH 8 1M Tris-HCl) for two times. DNA was eluted with elution buffer (1% SDS, 5mM EDTA, pH 8 1M Tris-HCl) for 30 minutes at room temperature. Supernatants were incubated with 200mM NaCl, proteinase K (20mg/ml) at 65^o^C for 4 hours. DNA was precipitated by ethanol 100% and washed with ethanol 70%. DNAI2 gene was analyzed by PCR using the following primers: forward 5’-TCCCATGGAAAGCTGTCTGG-3’ and reverse 5’- ATGGTGGGTGGATCAGTCTG-3’

**Genomic DNA extraction** - Genomic DNA of human cell lines were extracted by using QIAamp DNA Mini kit (Qiagen, Germantown, MD, USA) according to manufactured protocol

**Collection of specimens from lung cancer patients** - All human study has been conducted in accordance with the guidelines and regulations. The study using human specimens was approved by the Clinical Research Ethics Committee at National Cheng Kung University Medical Center (Tainan, Taiwan; IRB: A-ER-107-039). After surgical resection at National Cheng Kung University Hospital, specimens of patients with lung adenocarcinomas were collected for Immunohistochemical analysis or western blotting. The pathological data were analyzed by clinical pathologists. Informed consent was obtained from all subjects.

(1, 2)

**References**

1. Chen Y-C, Young M-J, Chang H-P, Liu C-Y, Lee C-C, Tseng Y-L, et al. Estradiol-mediated inhibition of DNMT1 decreases p53 expression to induce M2-macrophage polarization in lung cancer progression. Oncogenesis. 2022;11(1):25.

2. Wang Y-C, Wu Y-S, Hung C-Y, Wang S-A, Young M-J, Hsu T-I, et al. USP24 induces IL-6 in tumor-associated microenvironment by stabilizing p300 and β-TrCP and promotes cancer malignancy. Nature communications. 2018;9(1):3996.

3. Hung C-Y, Hsu T-I, Chuang J-Y, Su T-P, Chang W-C, Hung J-J. Sp1 in astrocyte is important for neurite outgrowth and synaptogenesis. Molecular neurobiology. 2020;57:261-77.

**(3)** **Supplementary Figure Legends**

**Suppl. Fig. 1. The global gene expression profile upon doxycycline-mediated induction of lung cancer with (Dox_1303) or without PTEN knockout by TAM injection (Dox_TAM_50) was studied by RNA-Seq. The distribution of gene expression levels is shown.**

**Supplementary Figure**


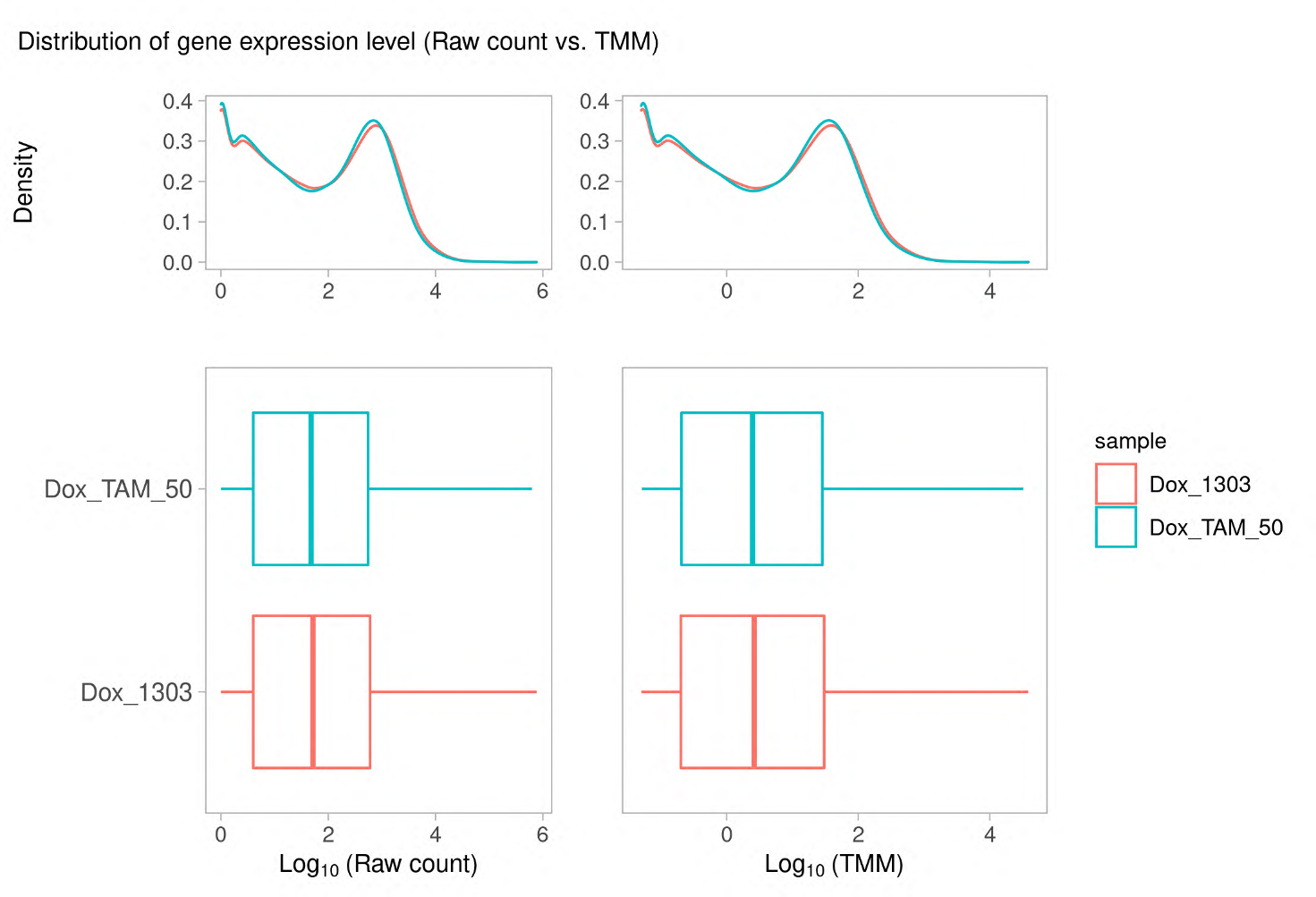
Suppl.Fig.1

**Suppl. Fig. 1.** The global gene expression profile upon doxycycline-mediated induction of lung cancer with (Dox_1303) or without PTEN knockout by TAM injection (Dox_TAM_50) was studied by RNA-Seq. The distribution of gene expression levels is shown.

Suppl.Fig.
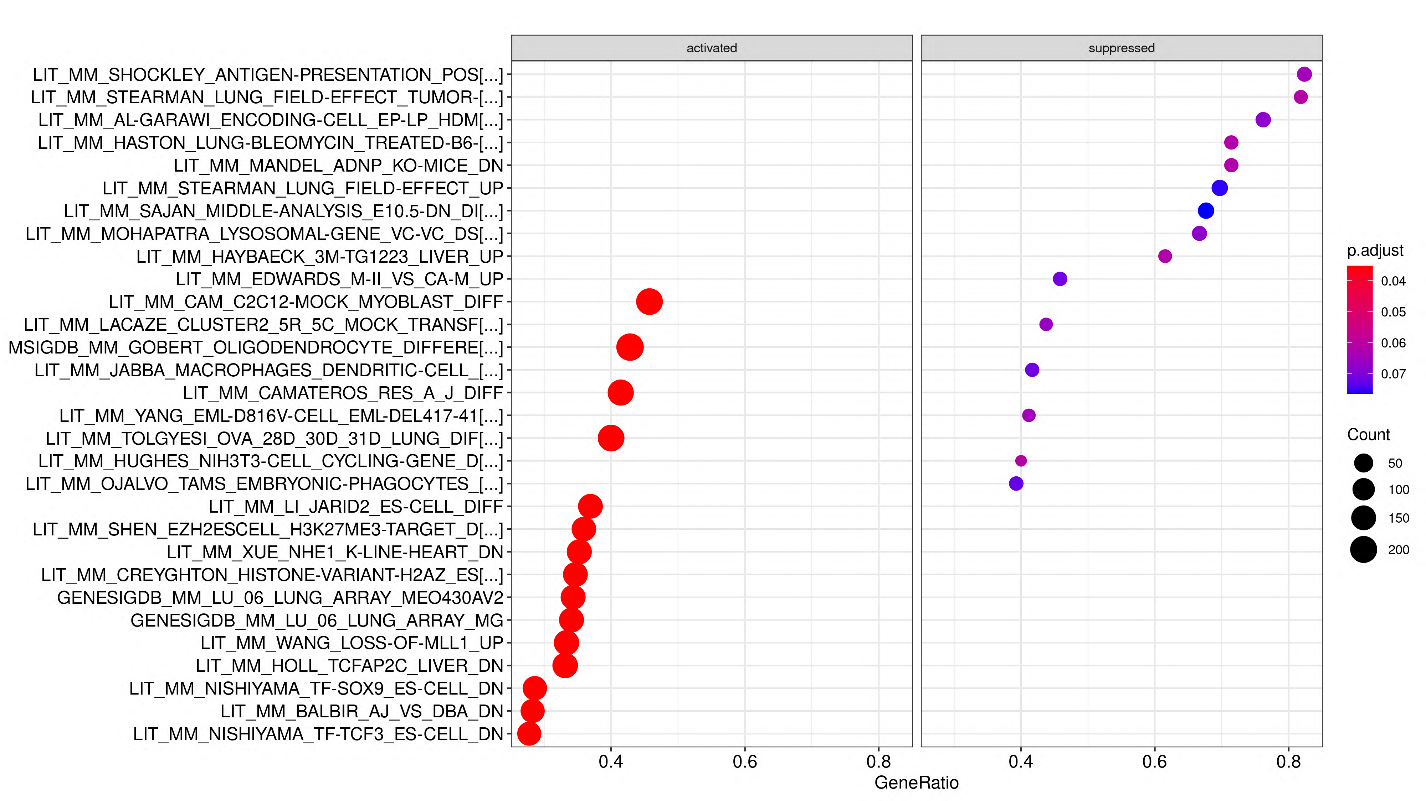
2

**Suppl. Fig. 2.** The global gene expression profile upon doxycycline-mediated induction of lung cancer with (Dox_1303) or without PTEN knockout by TAM injection (Dox_TAM_50) was studied by RNA-Seq. The enrichment of various gene expression-related various pathways was analyzed by GSEA.

**Suppl. Table 1a: Gene expression profile of cilium movement response in PTEN knockout mice.**

| Gene Symbol | Gene Name | log_2_ Fold Change* | P-value |
| --- | --- | --- | --- |
| Dnaic2 | Dynein axonemal intermediate chain 2 | 2.866062606 | 9.20E-07 |
| Hydin | HYDIN axonemal central pair apparatus protein | 2.864856585 | 1.66E-06 |
| Rsph4a | Radial spoke head 4 homolog A | 2.729489553 | 4.91E-06 |
| Wdr63 | WD repeat domain 63 | 2.668983046 | 3.02E-06 |
| Dnah5 | Dynein axonemal heavy chain 5 | 2.628868666 | 7.43E-13 |
| Dnah6 | Dynein axonemal heavy chain 6 | 2.61218878 | 1.97E-11 |
| Ak7 | Adenylate kinase 7 | 2.560044091 | 3.85E-09 |
| Spag16 | Sperm associated antigen 16 | 2.55371386 | 1.82E-06 |
| Dnali1 | Dynein axonemal light intermediate polypeptide 1 | 2.417974793 | 8.38E-07 |
| Dnah9 | Dynein axonemal intermediate chain 9 | 2.413086624 | 2.58E-06 |
| Ccdc40 | Coiled-coil domain containing 40 | 2.412862459 | 4.48E-07 |
| Dnah10 | Dynein axonemal heavy chain 10 | 2.263560729 | 2.94E-07 |
| Dnah3 | Dynein axonemal heavy chain 3 | 2.252559733 | 6.69E-05 |
| Cfap53 | Cilia and flagella associated protein 53 | 2.234515558 | 0.000107176 |
| Tekt1 | Tektin 1 | 2.204130879 | 6.67E-05 |
| Drc1 | Dynein regulatory complex subunit 1 | 2.193503243 | 7.45E-05 |
| Spef2 | Sperm flagellar 2 | 2.171886204 | 4.76E-05 |
| Nme5 | NME/NM23 family member 5 | 2.120344538 | 0.000164488 |
| Wdr66 | WD repeat domain 66 | 2.007010799 | 3.43E-06 |
| Cfap43 | Cilia and flagella associated protein 43 | 1.999807003 | 1.28E-09 |
| Wdr78 | WD repeat domain 78 | 1.805122765 | 7.64E-05 |

***=Log_2_(PTEN^-/-^/PTEN^+/+^)**

**Suppl. Table 1b: Gene expression profile related to axoneme, ciliary plasm, plasma membrane bounded cell projection cytoplasm in PTEN knockout mice.**

| Gene Symbol | Gene Name | log_2_ Fold Change* | P-value |
| --- | --- | --- | --- |
| Dcdc2a | Doublecortin domain containing 2a | 3.915524746 | 2.60E-05 |
| Dnaic2 | Dynein axonemal intermediate chain 2 | 2.866062606 | 9.20E-07 |
| Hydin | HYDIN axonemal central pair apparatus protein | 2.864856585 | 1.66E-06 |
| Iqca | IQ motif containing with AAA domain | 2.795382383 | 2.20E-05 |
| Rsph4a | Radial spoke head 4 homolog A | 2.729489553 | 4.91E-06 |
| Wdr63 | WD repeat domain 63 | 2.668983046 | 3.02E-06 |
| Dnah5 | Dynein axonemal heavy chain 5 | 2.628868666 | 7.43E-13 |
| Dnah6 | Dynein axonemal heavy chain 6 | 2.61218878 | 1.97E-11 |
| Spag16 | Sperm associated antigen 16 | 2.55371386 | 1.82E-06 |
| Tctex1d4 | Tctex1 domain containing 4 | 2.534186268 | 0.000208184 |
| Dnali1 | Dynein axonemal light intermediate polypeptide 1 | 2.417974793 | 8.38E-07 |
| Dnah9 | Dynein axonemal heavy chain 9 | 2.413086624 | 2.58E-06 |
| Ccdc40 | Coiled-coil domain containing 40 | 2.412862459 | 4.48E-07 |
| Drc3 | Dynein regulatory complex subunit 3 | 2.265079631 | 1.11E-07 |
| Dnah10 | Dynein axonemal heavy chain 10 | 2.263560729 | 2.94E-07 |
| Spag6l | Sperm associated antigen 6-like | 2.253480836 | 6.15E-05 |
| Dnah3 | Dynein axonemal heavy chain 3 | 2.252559733 | 6.69E-05 |
| Drc1 | Dynein regulatory complex subunit 1 | 2.193503243 | 7.45E-05 |
| Dnah12 | Dynein axonemal heavy chain 12 | 2.043528941 | 0.000142395 |
| Saxo2 | Stabilizer of axonemal microtubules 2 | 1.906550873 | 1.30E-04 |
| Wdr78 | WD repeat domain 78 | 1.805122765 | 7.64E-05 |
| Mapt | Microtubule-associated protein tau | 1.666033793 | 1.49E-09 |
| Traf3ip1 | TRAF3 interacting protein 1 | 1.572647644 | 3.01E-08 |

***=Log_2_(PTEN^-/-^/PTEN^+/+^)**

**Suppl. Table 1c: Gene expression profile related to axoneme part in PTEN knockout mice.**

| Gene Symbol | Gene Name | log_2_ Fold Change* | P-value |
| --- | --- | --- | --- |
| Dnaic2 | Dynein axonemal intermediate chain 2 | 2.866062606 | 9.20E-07 |
| Hydin | HYDIN axonemal central pair apparatus protein | 2.864856585 | 1.66E-06 |
| Wdr63 | WD repeat domain 63 | 2.668983046 | 3.02E-06 |
| Dnah5 | Dynein axonemal heavy chain 5 | 2.628868666 | 7.43E-13 |
| Dnah6 | Dynein axonemal heavy chain 6 | 2.61218878 | 1.97E-11 |
| Spag16 | Sperm associated antigen 16 | 2.55371386 | 1.82E-06 |
| Dnah9 | Dynein axonemal heavy chain 9 | 2.413086624 | 2.58E-06 |
| Dnah10 | Dynein axonemal heavy chain 10 | 2.263560729 | 2.94E-07 |
| Spag6l | Sperm associated antigen 6-like | 2.253480836 | 6.15E-05 |
| Dnah3 | Dynein axonemal heavy chain 3 | 2.252559733 | 6.69E-05 |
| Drc1 | Dynein regulatory complex subunit 1 | 2.193503243 | 7.45E-05 |
| Dnah12 | Dynein axonemal heavy chain 12 | 2.043528941 | 1.42E-04 |
| Saxo2 | Stabilizer of axonemal microtubules 2 | 1.906550873 | 1.30E-04 |
| Wdr78 | WD repeat domain 78 | 1.805122765 | 7.64E-05 |

***=Log_2_(PTEN^-/-^/PTEN^+/+^)**

**Suppl. Table 1d: Gene expression profile related to ciliary part in PTEN knockout mice.**

| Gene Symbol | Gene Name | log_2_ Fold Change* | P-value |
| --- | --- | --- | --- |
| Dcdc2a | Doublecortin domain containing 2a | 3.915524746 | 2.60E-05 |
| Dnaic2 | Dynein axonemal intermediate chain 2 | 2.866062606 | 9.20E-07 |
| Hydin | HYDIN axonemal central pair apparatus protein | 2.864856585 | 1.66E-06 |
| Iqca | IQ motif containing with AAA domain | 2.795382383 | 2.20E-05 |
| Cep126 | Centrosomal protein 126 | 2.755793754 | 8.79E-07 |
| Rsph1 | Radial spoke head 4 homolog A | 2.729489553 | 4.91E-06 |
| Rsph4a | Radial spoke head 4 homolog A | 2.729489553 | 4.91E-06 |
| Fam183b | Family with sequence similarity 183, member B | 2.712622422 | 1.25E-05 |
| Wdr63 | WD repeat domain 63 | 2.668983046 | 3.02E-06 |
| Dnah5 | Dynein axonemal heavy chain 5 | 2.628868666 | 7.43E-13 |
| Dnah6 | Dynein axonemal heavy chain 6 | 2.61218878 | 1.97E-11 |
| Spag16 | Sperm associated antigen 16 | 2.55371386 | 1.82E-06 |
| Tctex1d4 | Tctex1 domain containing 4 | 2.534186268 | 0.000208184 |
| Mlf1 | Myeloid leukemia factor 1 | 2.501374687 | 1.41E-05 |
| Dnali1 | Dynein axonemal light intermediate polypeptide 1 | 2.417974793 | 8.38E-07 |
| Dnah9 | Dynein axonemal heavy chain 9 | 2.413086624 | 2.58E-06 |
| Ccdc40 | Coiled-coil domain containing 40 | 2.412862459 | 4.48E-07 |
| Fam161a | Family with sequence similarity 161, member A | 2.320360632 | 2.10E-07 |
| Drc3 | Dynein regulatory complex subunit 3 | 2.265079631 | 1.11E-07 |
| Dnah10 | Dynein axonemal heavy chain 10 | 2.263560729 | 2.94E-07 |
| Spag6l | Sperm associated antigen 6-like | 2.253480836 | 6.15E-05 |
| Dnah3 | Dynein axonemal heavy chain 3 | 2.252559733 | 6.69E-05 |
| Drc1 | Dynein regulatory complex subunit 1 | 2.193503243 | 7.45E-05 |
| Spef2 | Sperm flagellar 2 | 2.171886204 | 4.76E-05 |
| Enkur | Enkurin, TRPC channel interacting protein | 2.100520413 | 0.000146136 |
| Dnah12 | Dynein axonemal heavy chain 12 | 2.043528941 | 0.000142395 |
| Mapk15 | Mitogen-activated protein kinase 15 | 2.042467745 | 9.22E-07 |
| Cfap126 | Cilia and flagella associated protein 126 | 2.001987689 | 2.25E-05 |
| Saxo2 | Stabilizer of axonemal microtubules 2 | 1.906550873 | 0.000130301 |
| Wdr78 | WD repeat domain 78 | 1.805122765 | 7.64E-05 |
| Map1b | Microtubule-associated protein 1B | 1.723525485 | 6.00E-10 |
| Cd24a | CD24a antigen | 1.687748889 | 1.42E-53 |
| Mapt | Microtubule-associated protein tau | 1.666033793 | 1.49E-09 |
| Cfap69 | Cilia and flagella associated protein 69 | 1.628186141 | 4.71E-05 |
| Traf3ip1 | TRAF3 interacting protein 1 | 1.572647644 | 3.01E-08 |
| Shank3 | SH3 and multiple ankyrin repeat domains 3 | 1.52652903 | 6.86E-10 |
| Prom1 | Prominin 1 | 1.288652676 | 2.36E-05 |

***=Log_2_(PTEN^-/-^/PTEN^+/+^)**

**Suppl. Table 1e: Gene expression profile related to microtubule-based movement in PTEN knockout mice.**

| Gene Symbol | Gene Name | log_2_ Fold Change* | P-value |
| --- | --- | --- | --- |
| Dnaic2 | Dynein axonemal intermediate chain 2 | 2.866062606 | 9.20E-07 |
| Hydin | HYDIN axonemal central pair apparatus protein | 2.864856585 | 1.66E-06 |
| Rsph4a | Radial spoke head 4 homolog A | 2.729489553 | 4.91E-06 |
| Wdr63 | WD repeat domain 63 | 2.668983046 | 3.02E-06 |
| Dnah5 | Dynein axonemal heavy chain 5 | 2.628868666 | 7.43E-13 |
| Dnah6 | Dynein axonemal heavy chain 6 | 2.61218878 | 1.97E-11 |
| Ak7 | Adenylate kinase 7 | 2.560044091 | 3.85E-09 |
| Spag16 | Sperm associated antigen 16 | 2.55371386 | 1.82E-06 |
| Kif19a | Kinesin family member 19A | 2.430845682 | 0.00013003 |
| Dnali1 | Dynein axonemal light intermediate polypeptide 1 | 2.417974793 | 8.38E-07 |
| Dnah9 | Dynein axonemal heavy chain 9 | 2.413086624 | 2.58E-06 |
| Ccdc40 | Coiled-coil domain containing 40 | 2.412862459 | 4.48E-07 |
| Ttc21a | Tetratricopeptide repeat domain 21A | 2.41260788 | 1.53E-06 |
| Dnah10 | Dynein axonemal heavy chain 10 | 2.263560729 | 2.94E-07 |
| Dnah3 | Dynein axonemal heavy chain 3 | 2.252559733 | 6.69E-05 |
| Cfap53 | Cilia and flagella associated protein 53 | 2.234515558 | 0.000107176 |
| Tekt1 | Tektin 1 | 2.204130879 | 6.67E-05 |
| Drc1 | Dynein regulatory complex subunit 1 | 2.193503243 | 7.45E-05 |
| Spef2 | Sperm flagellar 2 | 2.171886204 | 4.76E-05 |
| Nme5 | NME/NM23 family member 5 | 2.120344538 | 1.64E-04 |
| Dnah12 | Dynein axonemal heavy chain 12 | 2.043528941 | 1.42E-04 |
| Wdr66 | WD repeat domain 66 | 2.007010799 | 3.43E-06 |
| Cfap43 | Cilia and flagella associated protein 43 | 1.999807003 | 1.28E-09 |
| Wdr78 | WD repeat domain 78 | 1.805122765 | 7.64E-05 |
| Dynlrb2 | Dynein light chain roadblock-type 2 | 1.783776129 | 7.23E-06 |
| Map1b | Microtubule-associated protein 1B | 1.723525485 | 6.00E-10 |
| Mapt | Microtubule-associated protein tau | 1.666033793 | 1.49E-09 |
| Traf3ip1 | TRAF3 interacting protein 1 | 1.572647644 | 3.01E-08 |
| Kif21a | Kinesin family member 21A | 1.235467697 | 0.000178731 |

***=Log_2_(PTEN^-/-^/PTEN^+/+^)**

**Suppl. Table 1f: Gene expression profile related to Plasma membrane bounded cell projection cytoplasm in PTEN knockout mice.**

| Gene Symbol | Gene Name | log_2_ Fold Change* | P-value |
| --- | --- | --- | --- |
| Dcdc2a | Doublecortin domain containing 2a | 3.915524746 | 2.60E-05 |
| Dnaic2 | Dynein axonemal intermediate chain 2 | 2.866062606 | 9.20E-07 |
| Hydin | HYDIN axonemal central pair apparatus protein | 2.864856585 | 1.66E-06 |
| Ccdc113 | Coiled-coil domain containing 113 | 2.763750774 | 5.52E-06 |
| Cep126 | Centrosomal protein 126 | 2.755793754 | 8.79E-07 |
| Rsph4a | Radial spoke head 4 homolog A | 2.729489553 | 4.91E-06 |
| Wdr63 | WD repeat domain 63 | 2.668983046 | 3.02E-06 |
| Dnah5 | Dynein axonemal heavy chain 5 | 2.628868666 | 7.43E-13 |
| Ak7 | Adenylate kinase 7 | 2.560044091 | 3.85E-09 |
| Spag16 | Sperm associated antigen 16 | 2.55371386 | 1.82E-06 |
| Rsph1 | Radial spoke head 1 homolog | 2.50651385 | 1.74E-09 |
| Kif19a | Kinesin family member 19A | 2.430845682 | 0.00013003 |
| Dnali1 | Dynein axonemal light intermediate polypeptide 1 | 2.417974793 | 8.38E-07 |
| Ccdc40 | Coiled-coil domain containing 40 | 2.412862459 | 4.48E-07 |
| Ttc21a | Tetratricopeptide repeat domain 21A | 2.41260788 | 1.53E-06 |
| Iqcg | IQ motif containing G | 2.399527712 | 2.28E-05 |
| Foxj1 | Forkhead box J1 | 2.36063887 | 8.38E-19 |
| Fam161a | Family with sequence similarity 161, member A | 2.320360632 | 2.10E-07 |
| Spag6l | Sperm associated antigen 6-like | 2.253480836 | 6.15E-05 |
| Cfap53 | Cilia and flagella associated protein 53 | 2.234515558 | 1.07E-04 |
| Tekt1 | Tektin 1 | 2.204130879 | 6.67E-05 |
| Drc1 | Dynein regulatory complex subunit 1 | 2.193503243 | 7.45E-05 |
| Spef2 | Sperm flagellar 2 | 2.171886204 | 4.76E-05 |
| Nme5 | NME/NM23 family member 5 | 2.120344538 | 1.64E-04 |
| Mapk15 | Mitogen-activated protein kinase 15 | 2.042467745 | 9.22E-07 |
| Cfap126 | Cilia and flagella associated protein 126 | 2.001987689 | 2.25E-05 |
| Ubxn10 | UBX domain protein 10 | 2.000650837 | 3.05E-06 |
| Cfap43 | Cilia and flagella associated protein 43 | 1.999807003 | 1.28E-09 |
| Traf3ip1 | TRAF3 interacting protein 1 | 1.572647644 | 3.01E-08 |
| Gsn | Gelsolin | 1.115893778 | 9.10E-62 |

***=Log_2_(PTEN^-/-^/PTEN^+/+^)**

**Suppl. Table 1g: Gene expression profile related to motile cilium in PTEN knockout mice.**

| Gene Symbol | Gene Name | log_2_ Fold Change* | P-value |
| --- | --- | --- | --- |
| Dnaic2 | Dynein axonemal intermediate chain 2 | 2.866062606 | 9.20E-07 |
| Iqca | IQ motif containing with AAA domain | 2.795382383 | 2.20E-05 |
| Drc7 | Dynein regulatory complex subunit 7 | 2.7786175 | 5.31E-06 |
| Dnah5 | Dynein axonemal heavy chain 5 | 2.628868666 | 7.43E-13 |
| Ak7 | Adenylate kinase 7 | 2.560044091 | 3.85E-09 |
| Spag16 | Sperm associated antigen 16 | 2.55371386 | 1.82E-06 |
| Tctex1d4 | Tctex1 domain containing 4 | 2.534186268 | 2.08E-04 |
| Rsph1 | Radial spoke head 1 homolog | 2.50651385 | 1.74E-09 |
| Mlf1 | Myeloid leukemia factor 1 | 2.501374687 | 1.41E-05 |
| Dnali1 | Dynein axonemal light intermediate polypeptide 1 | 2.417974793 | 8.38E-07 |
| Dnah9 | Dynein axonemal heavy chain 9 | 2.413086624 | 2.58E-06 |
| Ccdc40 | Coiled-coil domain containing 40 | 2.412862459 | 4.48E-07 |
| Cfap65 | Cilia and flagella associated protein 65 | 2.41139332 | 1.01E-11 |
| Iqcg | IQ motif containing G | 2.399527712 | 2.28E-05 |
| Drc3 | Dynein regulatory complex subunit 3 | 2.265079631 | 1.11E-07 |
| Spag6l | Sperm associated antigen 6-like | 2.253480836 | 6.15E-05 |
| Drc1 | Dynein regulatory complex subunit 1 | 2.193503243 | 7.45E-05 |
| Spef2 | Sperm flagellar 2 | 2.171886204 | 4.76E-05 |
| Nme5 | NME/NM23 family member 5 | 2.120344538 | 1.64E-04 |
| Enkur | Enkurin, TRPC channel interacting protein | 2.100520413 | 1.46E-04 |
| Wdr66 | WD repeat domain 66 | 2.007010799 | 3.43E-06 |
| Cfap43 | Cilia and flagella associated protein 43 | 1.999807003 | 1.28E-09 |
| Saxo2 | Stabilizer of axonemal microtubules 2 | 1.906550873 | 1.30E-04 |
| Cfap69 | Cilia and flagella associated protein 69 | 1.628186141 | 4.71E-05 |

***=Log_2_(PTEN^-/-^/PTEN^+/+^)**

**Suppl. Table 1h: Gene expression profile related to axoneme assembly in PTEN knockout mice.**

| Gene Symbol | Gene Name | log_2_ Fold Change* | P-value |
| --- | --- | --- | --- |
| Dnaic2 | Dynein axonemal intermediate chain 2 | 2.866062606 | 9.20E-07 |
| Hydin | HYDIN axonemal central pair apparatus protein | 2.864856585 | 1.66E-06 |
| Rsph4a | Radial spoke head 4 homolog A | 2.729489553 | 4.91E-06 |
| Wdr63 | WD repeat domain 63 | 2.668983046 | 3.02E-06 |
| Dnah5 | Dynein axonemal heavy chain 5 | 2.628868666 | 7.43E-13 |
| Ak7 | Adenylate kinase 7 | 2.560044091 | 3.85E-09 |
| Spag16 | Sperm associated antigen 16 | 2.55371386 | 1.82E-06 |
| Rsph1 | Radial spoke head 1 homolog | 2.50651385 | 1.74E-09 |
| Dnali1 | Dynein axonemal light intermediate polypeptide 1 | 2.417974793 | 8.38E-07 |
| Ccdc40 | Coiled-coil domain containing 40 | 2.412862459 | 4.48E-07 |
| Iqcg | IQ motif containing G | 2.399527712 | 2.28E-05 |
| Spag6l | Sperm associated antigen 6-like | 2.253480836 | 6.15E-05 |
| Drc1 | Dynein regulatory complex subunit 1 | 2.193503243 | 7.45E-05 |
| Spef2 | Sperm flagellar 2 | 2.171886204 | 4.76E-05 |
| Cfap43 | Cilia and flagella associated protein 43 | 1.999807003 | 1.28E-09 |

***=Log_2_(PTEN^-/-^/PTEN^+/+^)**

**Suppl. Table 1.** The GO pathways significantly enriched in the differentially expressed genes between control mice and PTEN^-/-^ mice (Table 1a-1h).

| Gene  **Suppl.Table.2 The human and mouse primers sequences.** | Human | | Mouse | |
| --- | --- | --- | --- | --- |
|  | **F (5’-3’)** | **R (5’-3’)** | **F (5’-3’)** | **R (5’-3’)** |
| Pten | TGAGTTCCCTCAGCCGTTACCT | GAGGTTTCCTCT GGTCCTGGTA | TGAGTTCCCTCAGCCATTGCCT | GAGGTTTCCTCT GGTCCTGGTA |
| Nr2f1 | TGCCTCAAAGCCATCGTGCTGT | CAGCAGCAGTTTGCCAAAACGG | CCAACAGGAACT GTCCCATCGA | CCGTTTGTGAGT GCATACTGGC |
| Dnai2 | CCGCATTTGGTCTGAAGACAGC | GTTCCGTCCATCCTGGTGGTAA | GGACATTTCCAG AAAGGAGCAGC | TTGCGGTTGCAG GAGATGACGA |
| Dnai3 | CCACCTGAAAGAGTACCAGTCC | AAGAAAGTCGCACGGCTACCGA | CAAGCCGATAGA GGACTTCTGC | ACGGTGTAGAGGCTCACAGGTT |
| Muc5ac | CCACTGGTTCTATGGCAACACC | GCCGAAGTCCAGGCTGTGCG | CCACTTTCTCCTTCTCCACACC | GGTTGTCGATGCAGCCTTGCTT |
| Muc5b | CTGCTACGACAAGGACGGAAAC | AAGGCTGTGAGCGCACTGGATG | CTGAAGACCTGTCGGAACCCAA | GCCACACACTTCATCTGGTCCT |
| Dcdc2a | TGACAAGTCAACGATGAGAAGGC | CCAACTGTTGACTTAGAGTGGCG | GGAAAGGCTGAAGGAGACAACAG | TCTCTTGAGGGCTGGTGACTGT |
| Cep126 | CTCAGAAACTCCTCGAAGATCAAC | CATGCTCTGTAGCCTCAAGACTG | GCTCCAAGAGGTGTCAAGACTG | GCTTGCTGTTTATGGCAGTCAGA |
| Foxj1 | ACTCGTATGCCACGCTCATCTG | GAGACAGGTTGTGGCGGATTGA | CTCCTATGCCACTCTCATCTGC | GACAGGTTGTGGCGGATGGAAT |
| Dnah5 | CCACACCTTGATGAGAGCCATG | CAGTCCAGTCATTTGTGGCAACG | CAGAATCCACCATCGTCATGCG | TCAGGATAGCCTGCCTTGTCCA |
| Dnah6 | CCAATTCTGGTGATCTGTGGAGC | TACCCATAGCGCACTGTGTCAG | CCAATTCTGGTGATCTGTGGAGC | TACCCATAGCGCACTGTGTCAG |
| Spag16 | TCCAAGTCCTGGCAATGAGGTG | CACAACCGTGTGTGCCTCGTTT | GATCGTCTCTGGAAGATGGTGG | TGCTGTCACCACTTGAGGTAGC |
| Tctex1d4 | CCACGCTACAAGCTGGTATGCA | AAGAGCGAGGTGTTGGTGTAGG | GCACCCTCATACCGCTTAGAAC | CATAGTGCCTGCACCAGTTTCC |
| Hydin | GATCTCCTTCAGCTCTACCATCC | GGTAGGTCCAATGACACAGCCT | GGAGCATTGTTCCGATGTCTGAC | CTCTCCGTTTGGAGGCACAATC |
| Drc7 | CTCACCACCTATGAGGACTTGC | GCCAGGCTTGAAGTAGTCTGTC | TGAGAGCCTCTGGAACCACAAG | CTCCTCAGTCAAGGACAGGTGA |
| Kif19a | TGATCGCTCACATCAGTCCTGC | GCGATGTGGTAGGAGACGTTCA | GGAAGTCCAGATTGACACCTCC | CCTTCTCACTGTCCTCTTTGGTG |
| Gapdh | GTCTCCTCTGACTTCAACAGCG | ACCACCCTGTTGCTGTAGCCAA | CATCACTGCCACCCAGAAGACTG | ATGCCAGTGAGCTTCCCGTTCAG |

**Suppl. Table 2**. Primers were used in this study.
