# Supplementary figures and images for: PTEN decreases NR2F1 expression to inhibit ciliogenesis during EGFRL858R-induced lung cancer progression
Source: Cell Death Dis. 2024 Mar 18;15(3):225. doi: 10.1038/s41419-024-06610-z (PMC10948910; doi:10.1038/s41419-024-06610-z)

Fig.1C

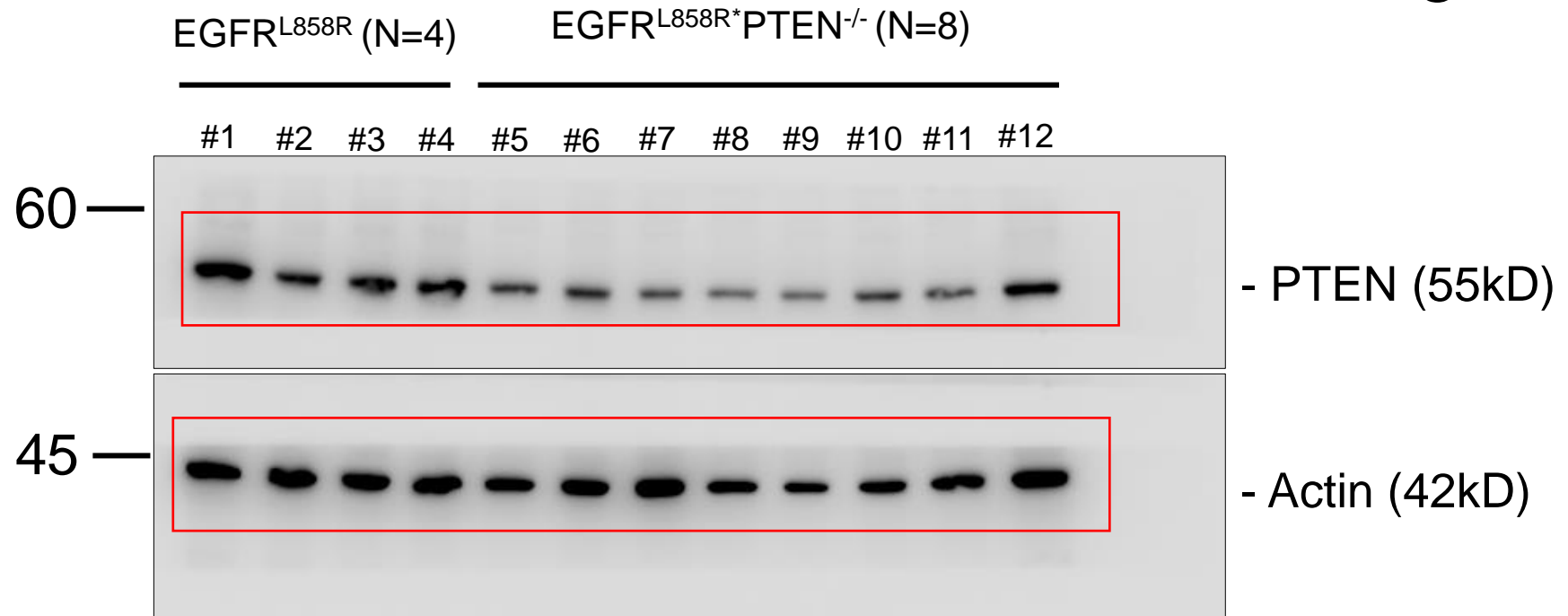

|          | Repeat 1 |   | Repeat 2 |   | Repeat 3 |   |
|----------|----------|---|----------|---|----------|---|
| scramble | +        | - | +        | - | +        | - |
| shPTEN   | -        | + | -        | + | -        | + |
| 75 —     |          |   |          |   |          |   |
| 60 —     |          |   |          |   |          |   |
| 45 —     |          |   |          |   |          |   |
|          |          |   |          |   |          |   |
| 60 —     |          |   |          |   |          |   |
| 45 —     |          |   |          |   |          |   |

- Actin (42kD)

Fig.4F

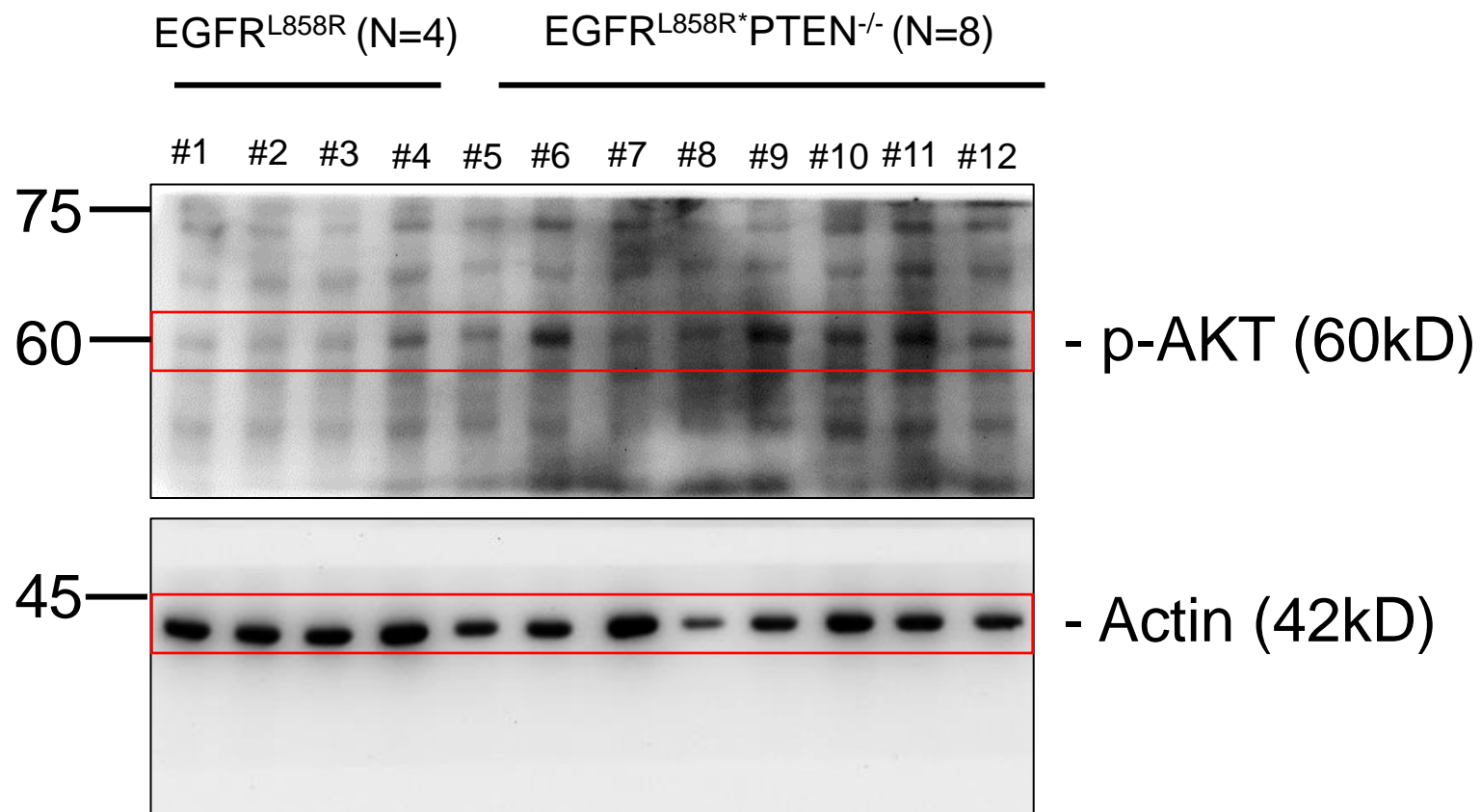

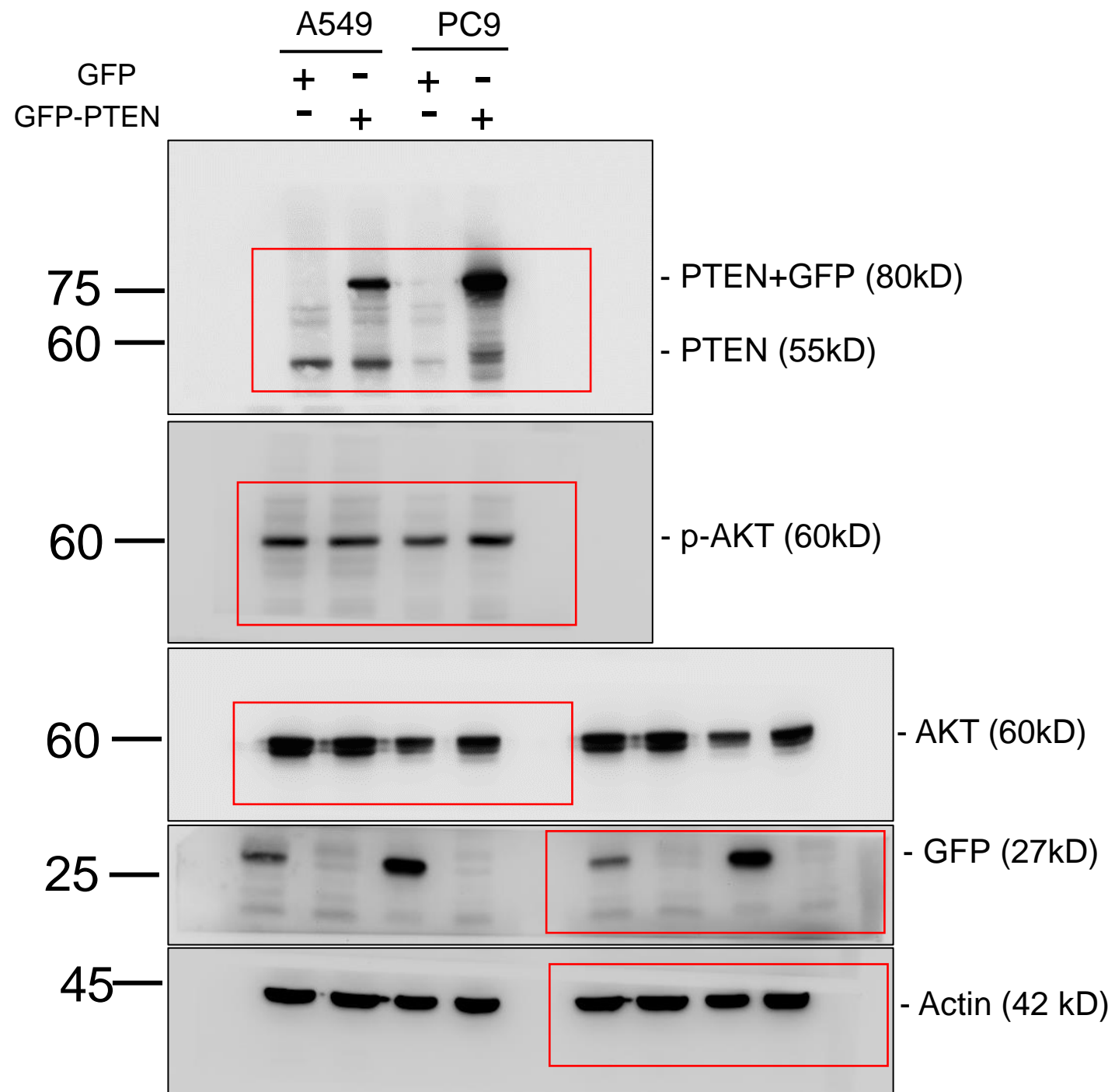

Fig.5B

Fig.5C  
(a)

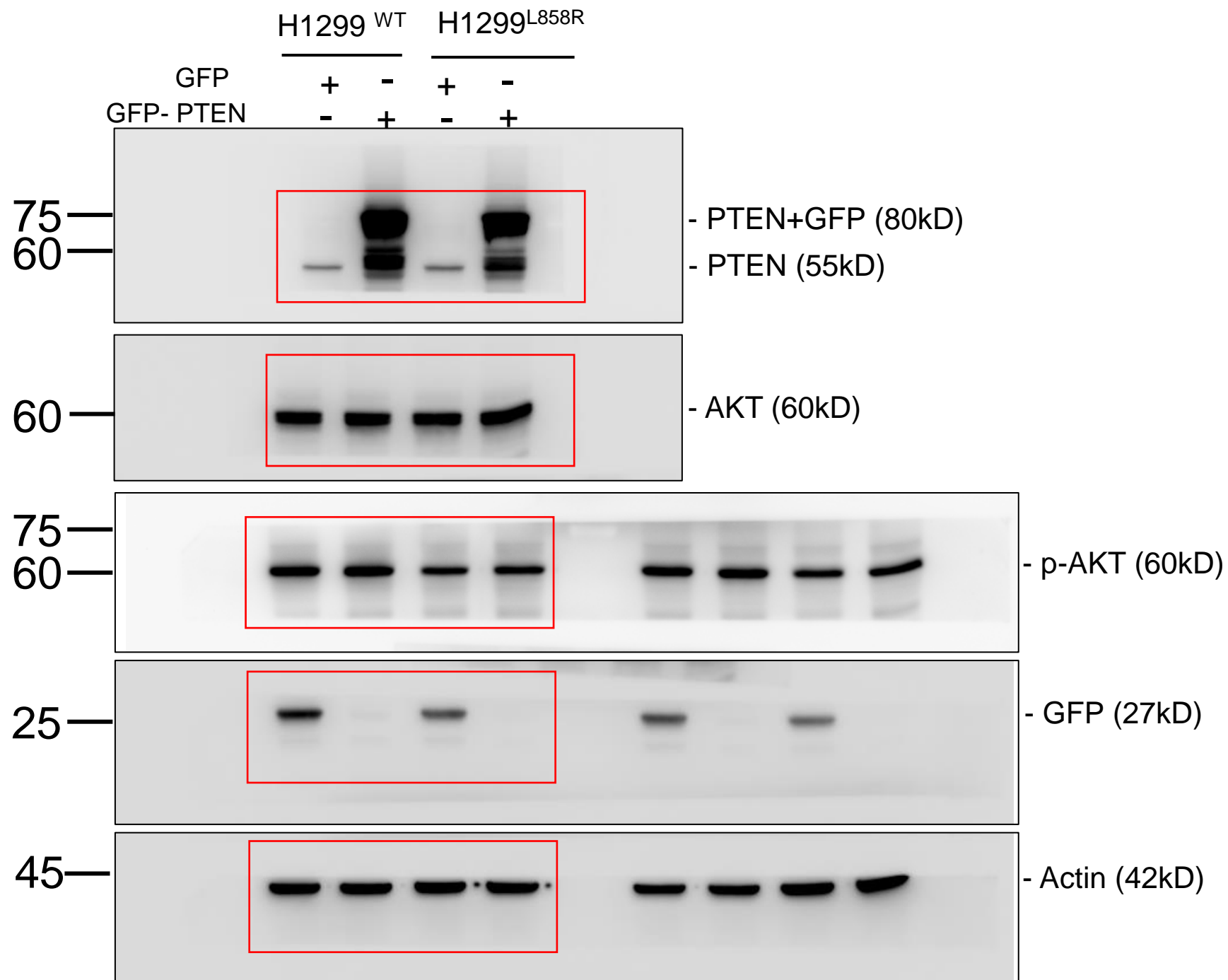

Supplement: Supplementary file 2 — Original Data File [file 41419_2024_6610_MOESM2_ESM.pdf]
